# A pre-post single-arm pilot trial of a digital multicomponent intervention for self-efficacy enhancement among online support groups of primary caregivers of children with neuro-muscular disorders: SEE-NMD protocol

**DOI:** 10.1371/journal.pone.0349543

**Published:** 2026-11-06

**Authors:** Nashit Irfan Aziz, Wardah Khalid, Sara Khan

**Affiliations:** 1 Brain and Mind Institute, Aga Khan University, Karachi, Sindh, Pakistan; 2 Department of Community Health Sciences, Aga Khan University, Karachi, Sindh, Pakistan; 3 Department of Medicine, Section of Neurology, Aga Khan University, Karachi, Sindh, Pakistan; IRCCS Medea: Istituto di Ricovero e Cura a Carattere Scientifico Eugenio Medea, ITALY

## Abstract

**Introduction:**

Spinal Muscular Atrophy (SMA) and Duchenne Muscular Dystrophy (DMD) are progressive genetic neuromuscular disorders that impose substantial caregiving demands on families. In Pakistan, where disease-modifying therapies remain largely inaccessible, care is predominantly supportive, frequently resulting in psychological distress and diminished caregiver confidence. An individual's belief in their capacity to execute required behaviours, self-efficacy, is a recognised determinant of caregiving quality, yet evidence on caregiver self-efficacy in SMA and DMD within low-resource settings remains sparse. This protocol describes the methods for an exploratory, single-arm, pre-post trial. The trial aims to generate preliminary evidence on changes in self-efficacy following a multicomponent, digitally delivered intervention and its implementation feasibility among primary caregivers of children with SMA and DMD in Pakistan.

**Methods and analysis:**

A single-arm, pre-post study design will recruit primary caregivers of children with SMA and DMD enrolled in the Treat-NMD Registry of Pakistan, with 40 caregivers per condition (80 total) analyzed as separate disease-specific cohorts. An eight-week intervention will be delivered via online support groups, comprising educational video clips, expert-led live sessions, pictorial guides, and progressively tapering audio reminders. The primary outcome, self-efficacy, will be measured using a culturally adapted, content-validated Urdu version of the DMD Caregiver Self-Efficacy Scale (DMD-CSES), assessed at baseline (T0) and eight weeks post-intervention (T1). Pre- and post-intervention scores will be compared using a paired t-test or Wilcoxon signed-rank test, depending on the data distribution, with analyses conducted in STATA Version 17.

**Ethics and dissemination:**

Ethical approval has been granted by the Ethics Review Committee of Aga Khan University (2025-11875-37040). Verbal informed consent will be obtained from all participants, and confidentiality will be maintained throughout the trial. The findings will be disseminated via peer-reviewed publications, conference presentations, and shared with the Treat-NMD registry network.

**Trial registration name and number:**

NCT07356063, Date of registration: 11/January/2026.

## Introduction

Spinal Muscular Atrophy (SMA) and Duchenne Muscular Dystrophy (DMD) are progressive, genetic neuromuscular disorders that substantially affect children and their families. SMA is characterized by degeneration of motor neurons in the spinal cord, resulting in progressive limb and respiratory muscle weakness [1], while DMD is an X-linked recessive disorder marked by progressive muscle degeneration and weakness [2].

The prevalence of SMA is 1–2 per 100,000 persons, and its incidence is estimated to be approximately 1 in 10,000 live births [3]. However, these pooled estimates are largely derived from older studies with small sample sizes and limited geographical scope, with an overrepresentation of European population. Similarly, the burden of DMD is often estimated using databases and registries. A comprehensive systematic review published in 2020 reported a pooled global prevalence of 7.1 (95% CI: 5.0–10.1) and a birth prevalence of 19.8 (95% CI: 16.6–23.6) per 100,000 live births [4]. In Pakistan, Bisma et. al analyzed 215 SMA patients from the country’s inaugural registry (MDR-PK) [5] and 130 DMD patients [6] from 2019–2024, suggesting that both conditions are present; however, neither study provides population-level incidence or prevalence estimates.

The economic burden of SMA and DMD is substantial worldwide, though almost entirely characterized in high-income settings. A systematic review pooling 55 studies across 32 countries estimated a weighted average annual per-patient cost of SMA at US$109,906, with caregivers logging nearly 3,000 hours of caregiving annually [7]. A comparable systematic review of DMD across 14 countries reported direct non-medical costs (home modifications, mobility aids, and similar) reaching as high as US$103,800 per patient annually [8]. To our knowledge, no published data quantify the financial burden of SMA or DMD on families in Pakistan, where caregivers already face the added strain of managing these conditions without the disease-modifying treatments, multidisciplinary care networks, or social support systems. These demands may contribute to social isolation, financial strain, and psychological distress among caregivers, as well as diminished confidence in their ability to manage caregiving [9]. Thus, understanding this level or ‘self-efficacy’ is of critical importance. Self-efficacy, by definition is as an individual’s belief in their capacity to execute behaviours required to achieve desired outcomes [9]. It is a recognised predictor of caregiver stress and burnout in chronic paediatric diseases: those with higher self-efficacy approach caregiving with more confidence and report lower burnout, while lower self-efficacy is linked to helplessness, anxiety, and depression, potentially compromising both caregiver wellbeing and child’s quality of care [10]. Despite caregiver burden, evidence investigating a caregiver’s self-efficacy for neuromuscular conditions remain limited. Across the literature, only a single study has validated a self-efficacy scale for caregivers of DMD patients [11], and another reported general self-efficacy levels among parents of children with SMA with a mean score of 46.18 ± 9.26 on the General Self-Efficacy Scale (GSES), where observed scores ranged from 33 to 73 [12]. A recent scoping review of psychosocial interventions for neuromuscular disease caregivers confirms that this evidence gap is condition-specific rather than incidental [13].

Support groups may offer caregivers’ opportunities to share their lived experiences, enhance their disease-related knowledge, and access emotional support from individuals facing similar challenges [14]. However, the concept is also in its infancy in Pakistan, with no formal organisations targeting neuromuscular disorders. Furthermore, conventional in-person support groups may be even more inaccessible due to geographical barriers, time constraints, or transportation difficulties. Online (internet-based) support groups have therefore emerged as a practical alternative in literature [15,16]. Such platforms also facilitate peer-to-peer interaction, professional guidance where available, and access to relevant educational resources from within the home environment.

Evidence from caregiver-focused interventions in neurodevelopmental disabilities suggests that structured parent training programmes can significantly improve parental self-efficacy, with a meta-analysis reporting a standardized mean difference of 0.60 (95% CI: 0.38–0.83) relative to the baseline [17]. More specifically, a trial of digital peer support for caregivers of neuromuscular conditions demonstrated good feasibility and acceptability [18] and telemedicine services for neuromuscular diseases more broadly have been shown to improve continuity of and access to care for patients and caregivers alike [19,20]. These findings suggest the potential applicability of such interventions to caregivers, even within resource-limited health systems, which remain largely oriented toward acute rather than chronic care [21].

Due to a dearth of structured interventions specifically designed for SMA and DMD, both globally and in Pakistan, this trial will be evaluating the changes in self-efficacy in primary caregivers of children with SMA or DMD, following an 8-week, digitally delivered, multicomponent intervention. In addition, it is intended to assess the feasibility of delivering the intervention by examining the participant recruitment, retention, attendance, and satisfaction. Reporting the protocol in advance of trial completion allows for transparent, pre-specified documentation of the trial’s methods, and supports comparison between planned and executed procedures upon its completion.

## Materials and methods

### Study design and setting

This study will be a single-arm pre–post interventional pilot trial conducted digitally on caregivers of patients enrolled in the Treat-NMD Registry of Pakistan (previously also called the Muscular Dystrophy Registry of Pakistan) [22]. Treat-NMD Registry of Pakistan is the national subdivision of Treat-NMD Global Registry Network (a federated network of 67 independent patient registries that collects high-quality information on rare Neuro-Muscular Dystrophy (NMD) patients worldwide [23]. Treat-NMD Pakistan is established through a systematic ascertainment of patients, where every patient suspected of having DMD or SMA nationwide is referred to the Aga Khan University Hospital (AKUH) for confirmatory genetic testing. Following genetic confirmation, patient details are identified from the AKUH laboratory records and enrolled via telephonic consent (self, family, friend) into the registry. Registry enrollment is therefore based on diagnostic confirmation rather than a voluntary registration initiative.

Since SMA and DMD are rare disorders, recruitment for this trial is anticipated to be difficult, owing to a relatively small pool of eligible and consenting caregivers. Allocating caregivers to a control group could further reduce the number of available participants. Therefore, rather than proceeding directly to a resource-intensive controlled trial, this study is intended as an exploratory design.

SMA and DMD differ in disease course, typical caregiving demands and treatment landscape, and the primary outcome instrument (DMD-CSES) was originally developed for DMD caregivers. For these reasons, the trial is designed as two parallel (disease-specific) cohorts, rather than a pooled one. Each cohort has its own sample size and analysis, and no formal comparison between them is planned.

The reporting of this protocol follows the SPIRIT 2025 Statement [24] (S1 Checklist, Fig 1) for interventional trials and the TIDieR checklist [25] (S2 Checklist) for intervention descriptions. The completed checklists for both are provided as supplementary files (S1 Checklist and S2 Checklist, respectively).

**Fig 1:**
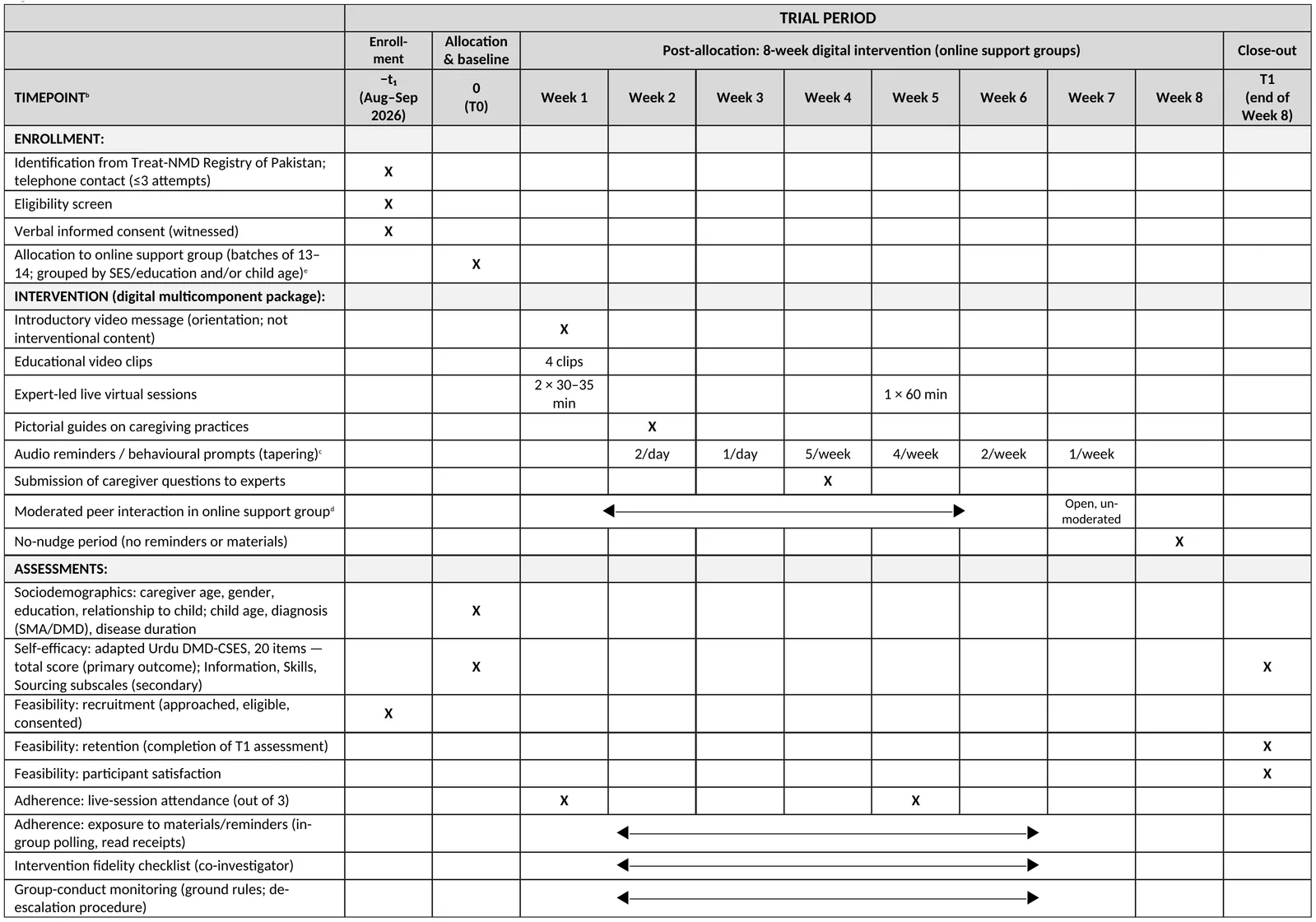
SPIRIT – Enrollment schedule.

All trial participants will be administered the questionnaire at two points: T0 (baseline), and T1 (8 weeks after intervention), to assess change in study outcome (self-efficacy), as demonstrated in (Fig 2).

**Fig 2:**
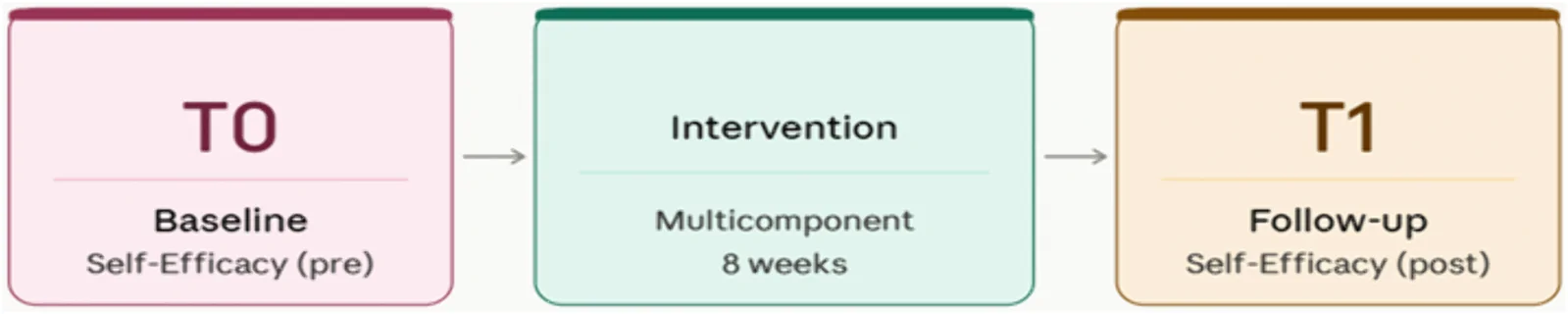
Outline of the proposed trial.

### Sample size

Sample size calculation was performed using G*Power software version 3.1.9.7 based on a two-tailed t-test with a significance level of 0.05 and a desired power of 80%. We also conducted an extensive literature review to identify an anchor-based minimally important clinical difference (MCID) for caregiver self-efficacy in these rare conditions; however, no such evidence was available. Thus a 0.5 standard deviation criterion was selected as a pragmatic, distribution-based estimate rather than a disease-specific clinically established threshold. Using these parameters, we calculated an estimated sample size of 34 participants. After adjusting for a 15% anticipated dropout rate, the sample size was inflated to 40, for each condition (SMA, DMD). However, we acknowledge that this sample size estimate is preliminary and is based on limited data. The data collected from this trial can be used to calculate a more accurate effect size, which will inform future sample size calculations for larger-scale trials [26]. This approach aligns with the recommendations for iterative sample size refinement in the absence of strong prior evidence [27,28].

### Participants

These will be the primary caregivers. Primary caregivers are the individuals (from family) who provide most of the care and support for the child with SMA or DMD [11]. The eligibility criteria for the trial will be as follows:

Inclusion:

1. Primary caregiver of a child diagnosed with SMA or DMD.
2. Access to a smartphone (or similar device) with Internet connectivity.

Exclusion:

1. Caregivers who are unable to understand the language of communication (Urdu).
2. Caregivers of children who have been diagnosed for less than one year.
3. Caregivers who are currently participating in other support group interventions.

### Recruitment

Participants will be recruited using a non-probability purposive sampling approach from the Treat-NMD Pakistan registry. Eligible participants will include those with a minimum of one year of caregiving experience at the time of recruitment. This threshold ensures that participants have transitioned past the initial ‘turbulent’ phase of diagnosis into a more established caregiving role. This approach is supported by evidence indicating that caregiver burden often stabilizes after the first year. For instance, Klietz et al. observed no significant fluctuations in Parkinson’s caregiver burden between the one-year mark and subsequent follow-ups [29]. Furthermore, recent reviews have identified a stable average trajectory of burden as the most prevalent long-term pattern [30], suggesting that experience beyond 12 months provides a more consistent baseline for intervention.

Recruitment will be conducted between August-September 2026 by trained recruiters (research associates/study investigators). Caregivers listed in the Treat-NMD registry will be approached for participation, and recruitment will be conducted sequentially in the year of diagnosis, beginning with earlier records and progressing to more recent entries.

Participants will be contacted via telephone. Up to three contact attempts spaced overtime will be made for each caregiver. Individuals who do not respond after three attempts will be classified as non-responders and will not be pursued further unless they initiate contact with the research team. Eligibility screening will be conducted during the call using the predefined criteria. Caregivers who meet the eligibility criteria will be provided with brief information about the trial, including its timeline and expected commitment, and will be invited to participate. Those expressing willingness to participate will be provided with a detailed explanation of the procedures, after which verbal informed consent will be obtained. Caregivers who give consent will be considered officially enrolled in the trial. Following enrollment, participants will be allocated to online support groups in batches of 13–14 individuals.

### Intervention

The intervention was developed by a team of internal experts (for the trial, referred as such for their help with the modifying the intervention’s content and self-efficacy scale). The team included a neuromuscular specialist, a paediatric neurologist, a physiotherapist, an occupational therapist, a dietician, a speech therapist and two neuroepidemiologists. The intervention was designed as a digitalised multicomponent package to enhance self-efficacy among primary caregivers of children with SMA and/or DMD. It includes three core components: (i) asynchronous educational materials (video clips and pictorial guides), (ii) live sessions, and (iii) scheduled audio-based behavioural prompts.

This intervention will be delivered over 8 weeks in batch-wise groups. Group composition will be guided by key participant characteristics, including either socioeconomic status or educational level of caregivers or/and the age of the care recipient/patient, such that participants with broadly similar profiles on these parameters are grouped together, to facilitate effective interaction and engagement during the intervention delivery. The intervention will include three live sessions in total: two in Week 1 (30–35 minutes each) and one in Week 5 (60 minutes), while educational content and reminders will be delivered throughout the intervention period with decreasing frequency to promote gradual autonomy to participants. Intervention fidelity will be monitored by a co-investigator (NIA), using a checklist. Live sessions will follow standardized scripts and slide decks to ensure consistency. After delivering the asynchronous educational materials and viewing “seen” receipt by all participants on each online support group, they will be asked at least twice, if they had viewed the material. Reminder prompts will be disseminated through online support groups and viewing will similarly be confirmed via in-group polling, in addition to the application's message delivery/read indicators where available.

A summary table of this Intervention is given in (Fig 3):

**Fig 3:**
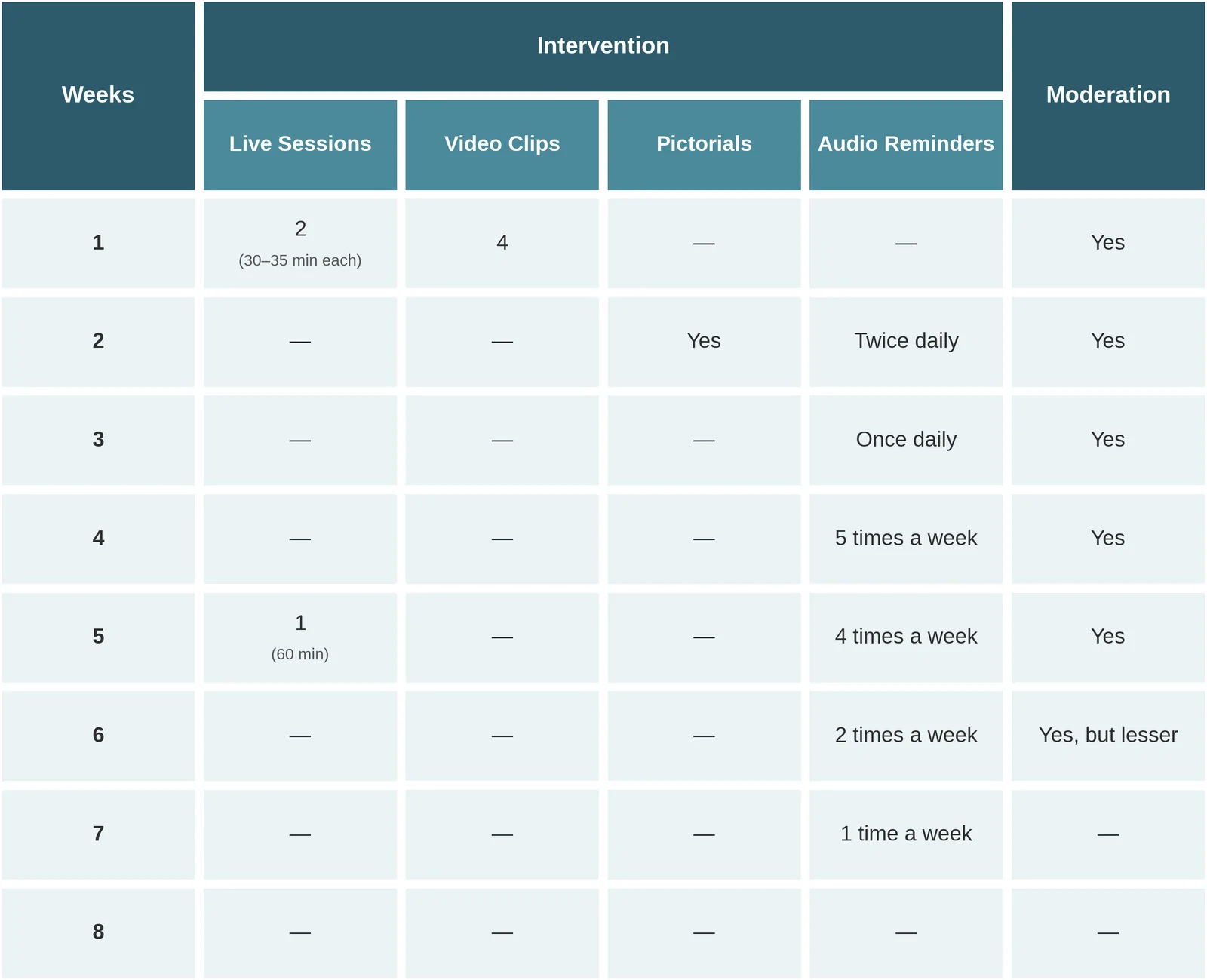
Summary table of a digital multicomponent intervention.

A detailed description of the plan of this intervention’s delivery, by weeks, is outlined as follows:

Week 1: At the start of Week 1, participants (caregivers) will receive an Introductory video message outlining the multi component intervention plan. The introductory video is not part of the interventional content. This will be followed by 2 video clips, providing practical demonstrations on disease understanding and rehabilitation, on the same day. After a day’s gap, participants will attend a live session with the experts, lasting 30–35 minutes, where a group of experts will cover the same content delivered in the video clips, to reinforce their message. The following day, 2 more video clips will be shared with the participants, demonstrating solutions to further aspects of disease rehabilitation. The next day, participants will receive another live session with the experts, lasting 30–35 minutes, revising the materials shared on the video clips. Thus, this week will provide a continuous revision on foundational education on disease and its caregiving principles.

Week 2: In Week 2, participants will receive pictorial guides covering key caregiving practices. In addition, the online support groups will deliver twice-daily audio reminders, which will act as behavioural cues to facilitate the translation of knowledge and learned skills into practice.

Week 3: In Week 3, participants will receive one audio reminder daily. Prompts will encourage caregivers to rewatch previously shared video clips and apply them.

Week 4: In Week 4, participants will receive a total of five audio reminders during the week. These will encourage continued application of caregiving practices. Reminders will be delivered irrespective of prior task completion to ensure consistent exposure to behavioural cues. Participants will also be invited to submit questions or concerns to experts in preparation for the Week 5 session.

Week 5: A third expert-led virtual session (of approximately 60 minutes) will be conducted to address major questions related to the application of caregiving practices over the preceding four weeks. While core content will remain standardized, the session will incorporate participant queries.

Participants will also be encouraged to engage with and support one another during the session.

During Week 5, participants will receive four reminder messages, reinforcing key caregiving behaviours.

Weeks 6: The support groups will remain open for moderated peer interaction. Reminder frequency will be reduced to two audio messages per week, delivered at the beginning and end of the week, to reinforce continuity of caregiving practices while promoting increasing independence.

Week 7: The platform will remain open for participant interaction without active moderation.

A final audio reminder will be delivered at the end of the week, encouraging sustained caregiving practices beyond the intervention period.

Week 8: In Week 8, the intervention will be stopped, and no reminders or educational materials will be delivered. This no-nudge period will allow participants to independently maintain learned behaviours without external prompts.

### Outcome

The primary outcome for analysis will be the change in total self-efficacy score between baseline (T0) and post-intervention (T1). Subscale scores (Information, Skills, Sourcing) within the scale, and a few feasibility measures (participant recruitment, retention, attendance, and satisfaction), will be analyzed as secondary outcomes.

Intervention adherence will be quantified using descriptive metrics: (1) live session attendance, as the proportion of sessions attended out of 3 (two in Week 1 and one in Week 5) total; (2) educational material exposure, as the percentage of video clips and pictorial guides confirmed as viewed via in-group polling.

The research team undertook a structured mapping process to ensure that self-efficacy scores and the intervention was contextually appropriate and conceptually aligned with the caregiving realities of families managing Duchenne Muscular Dystrophy (DMD) and Spinal Muscular Atrophy (SMA) patients in Pakistan’s local setting. Self-efficacy was measured by adapting the Duchenne Muscular Dystrophy Caregiver Self-Efficacy Scale (DMD-CSES), a psychometrically validated instrument originally developed for caregivers of individuals with DMD in Turkey [11]. The DMD-CSES is a self-administered questionnaire that assesses caregiver confidence across three core domains: Information (knowledge and understanding of the disease and caregiving requirements), Skills (ability to implement caregiving practices such as nutrition, rehabilitation, and symptom management), and Sourcing (ability to access external support systems, including healthcare services, financial resources, and time management). It consists of 18 items rated on a 5-point Likert scale ranging from strongly disagree to strongly agree, and total scores are calculated by summing all responses, with higher scores indicating higher caregiver self-efficacy. The adapted instrument retained the original three-domain conceptual framework of DMD-CSES but comprised 20 items following expert-recommended separation of double-barreled items to improve clarity and content coverage. This trial is not intended to establish the full psychometric properties of the adapted instrument. Adaptation and content validation were undertaken to ensure conceptual relevance, prior to its use as an outcome measure. Comprehensive psychometric validation, including construct validity, factor structure, and test–retest reliability, would require a larger independent validation study and was therefore beyond the objectives of this trial.

To start the adaptation process (and ensure that the intervention’s design content appropriately targeted the same domains as DMD-CSES), input was initially sought from internal experts (Step 1). These experts contributed targeted feedback, ensuring that it was in an understandable vocabulary for caregivers of varying literacy levels and that each item put in the scale reflected realistic caregiving expectations in Pakistan. Their opinions were based on clinical experiences in respective fields, to account for the differences in healthcare access, rehabilitation availability, caregiver support structures, and cultural caregiving roles between Turkey with Pakistan. Following contextual modifications, the scale was translated into Urdu language, using independent forward and backward translation (Step 2).Thereafter, a few caregivers participated in cognitive debriefing for improving scale’s comprehensibility and appropriateness within the target participants (Step 3). This feedback was gathered through in-depth interviews (IDIs), effectively excluding them from trial enrollment. Simultaneously, these caregivers also helped in making final modifications to the Intervention’s design, to ensure its educational content, terminology, and delivery format was aligned with our population needs, norms and feasibility.

Following participants’ feedback, the scale, was sent to a panel of external experts for content validation process. These experts were referred as external experts as they were not involved in the scales or the interventions initial designing phase. The panel comprised nine experts, including epidemiologists, neurologists, biostatisticians, mental health professionals, rehabilitation specialists, and nurses. Content validation involved systematic ratings for each item of the scale, to assess relevance and clarity, and was quantified using the Content Validity Index (CVI) (Step 4). The external experts’ feedback was incorporated by the co-investigators (NIA, WK) to produce a final English and Urdu version of the scale (Step 5). The summary of steps for scale adaptation is better illustrated in (Fig 4). The finalized English and Urdu versions of the outcome’s scale were completed before participant recruitment and will be used consistently throughout the trial. Internal consistency of the adapted scale will be assessed following completion of baseline data collection using Cronbach's alpha, calculated separately for the DMD and SMA subgroups.

**Fig 4:**
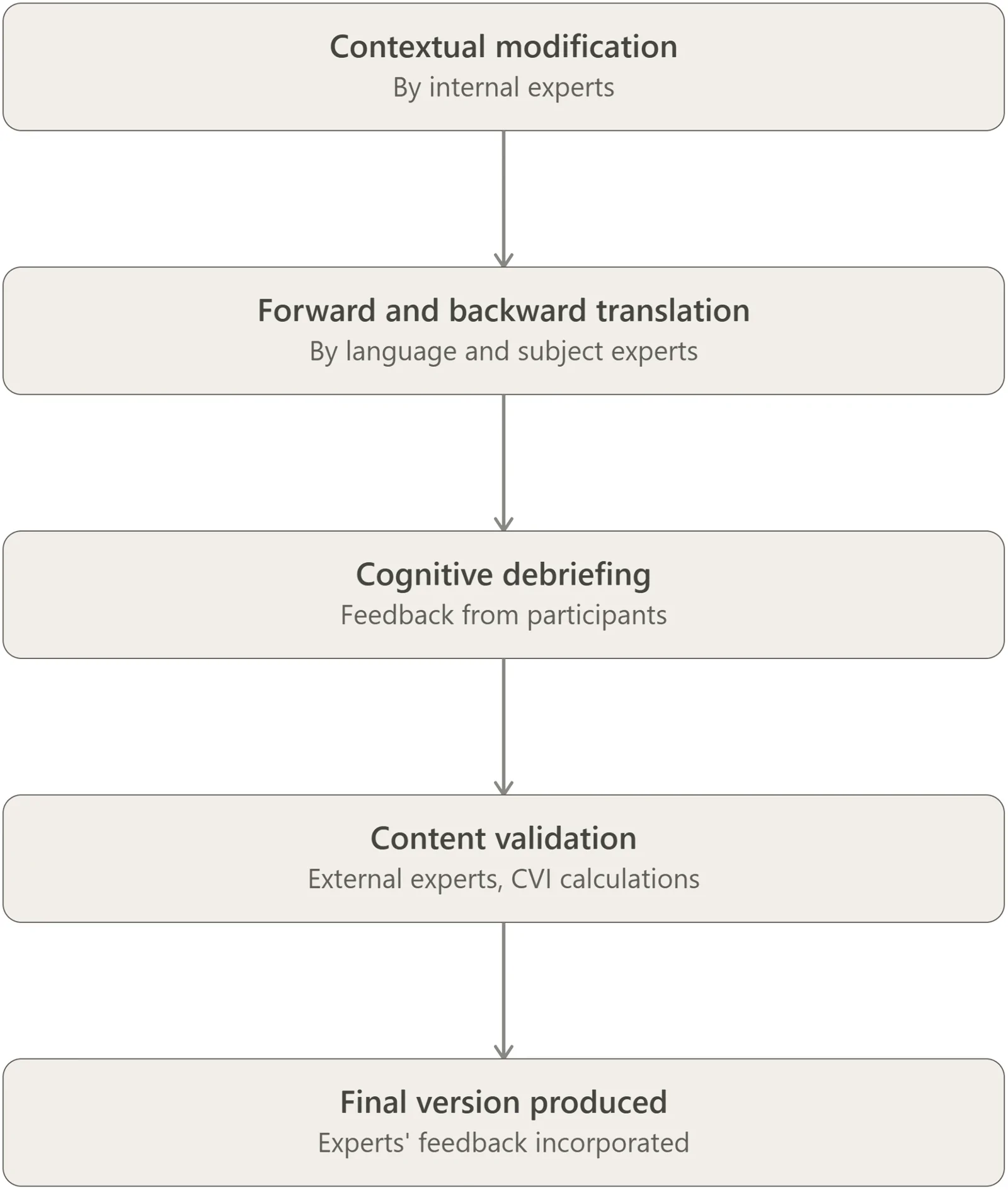
Summary of scale adaptation.

### Data management

All data will be collected and stored electronically and will only be accessible to authorized personnel. Since the registry has participants from all over Pakistan, the data collection would be conducted via calls. These calls will be conducted by trained study staff. The staff will obtain a verbal consent which will be documented along with a witness. The consent will explain the trials’ purpose, procedures, potential risks and benefits, and the right to withdraw from the study at any time without penalty. Participants’ identities will be protected using pseudonyms or code numbers.

During the live sessions and in unmoderated peer interactions, there is a minimal risk of heated discussion. To reduce this, participants will be given a set of rules to follow at the start of the Intervention (do's and don’ts). These will include guidance on respectful communication, maintaining confidentiality of other participants’ information, avoiding the exchange of medical advice or health-related misinformation between participants, and protecting the personal information of all group members. Participants will also be advised that private consultations with the study experts outside the designated Q&A sessions will not be available, given the limited-contact nature of the online group format. In the event of repeated inappropriate behaviour, a de-escalation approach will be followed: initial private communication with the participant concerned, a reminder of group rules, temporary moderation of their participation, and, if behaviour persists, removal from the group. Experts added in the online support groups are medical/allied health care professionals, equipped with counselling experience on handling such risks. Similarly, during live sessions or other proctored support group activities, the host controls will be strict (for example muting or disabling participants from cross-talking).

### Data analysis

Data will first be reviewed for completeness and consistency. Cleaning procedures will include range checks, detection of outliers, and validation of logical consistency across related variables. SMA and DMD caregivers constitute two independent cohorts, each analyzed under its own primary analysis; data from the two conditions will not be pooled or formally compared. Within each condition, the primary analysis of change in self-efficacy will include all participants with paired baseline and post-intervention CSES scores; participants missing either assessment will be excluded from that condition's paired comparison but retained in descriptive and feasibility reporting, with reasons for withdrawal or missing data described transparently. No imputation for missing baseline or post-intervention data is planned, given each condition's small sample size.

Descriptive statistics will be used to summarize the sociodemographic characteristics of participants, including caregiver age, gender, education level, relationship to the child, and child’s age, diagnosis, and disease duration. Continuous variables (e.g., caregiver age) will be presented using mean and standard deviation (SD) if normally distributed, or median and interquartile range (IQR) if not. Categorical variables (e.g., gender) will be summarized using frequencies and percentages.

Before inferential analysis, the distribution of total self-efficacy scores and subscale scores will be assessed for normality using histograms, Q-Q plots, and the Shapiro-Wilk test. To evaluate the change in self-efficacy following intervention, pre and post self-efficacy scores will be calculated. If the difference scores (post-pre) are normally distributed, a paired t-test will compare pre- and post-intervention self-efficacy scores. If normality is not met, the Wilcoxon signed-rank test will be applied. The mean paired difference (or median paired difference, where appropriate) will be reported together with 95% confidence intervals. Effect sizes (Cohen's d) for paired samples will also be calculated to quantify the magnitude of change following the intervention.

All statistical tests will be two-sided, with significance defined as p < 0.05. Analyses will be conducted using STATA Version 17.

### Ethical considerations

Ethical approval was taken from the Ethics Review Committee (ERC) of Aga Khan University (2025-11875-37040), and the trial was prospectively registered on clinicaltrials.gov by the Principal Investigator (NCT07356063), before recruitment. In accordance with ERC’s SOPs, verbal informed consent will be obtained and appropriately documented with a co-signed witness. Written informed consent will not be feasible because participants are geographically dispersed across Pakistan. Formal independent trial monitoring and Data Monitoring Committee (DMC) are not required for this trial. Given its exploratory nature with a small sample size and a short intervention duration, no interim analyses or stopping rules are planned. The intervention is non-pharmacological and poses minimal risk to participants; identified risks are mitigated through pre-specified strategies outlined above. Thus, trial conduct, protocol adherence, data quality, and ethical compliance will be overseen directly by the principal investigators throughout the study. All participant information will be treated as strictly confidential, and no identifying data will be disclosed in any publication or presentation.

## Discussion

This protocol describes a single-arm, pre–post evaluation of a structured, digitalised intervention designed to enhance caregiving self-efficacy in parents of children with SMA and DMD, in online support groups. The intervention is theory-based and targets key domains of caregiving confidence through education, skills reinforcement, and peer-supported engagement.

While this trial will address an important gap, several limitations warrant discussion. The relatively small sample size limits its statistical power and generalisability, and findings would be interpreted as exploratory. Additionally, without a comparator group, observed changes in self-efficacy will not be attributed exclusively to the intervention, as external influences including Hawthorne effects, temporal effects or regression to the mean may contribute. Another limitation is potential social-desirability bias (caregivers may overstate confidence), as we will rely on interviewer-administered self-report measures. Despite planned grouping, based on disease-related and socioeconomic characteristics, heterogeneity in engagement, uptake, and perceived benefit may persist between groups. Variability in how participants interact with different components of the intervention may influence observed outcomes. Future studies should incorporate these measures to provide a more comprehensive evaluation of the intervention’s feasibility. Similarly, a full psychometric validation (construct validity, factor analysis, and test-retest reliability) was out of the trial’s scope and should be established through future dedicated validation studies involving larger sample sizes. As the DMD-CSES was originally developed and validated for DMD caregivers, conclusions regarding the scale's validity and performance among SMA caregivers in particular will remain preliminary, pending dedicated psychometric evaluation in this subgroup.

Despite these limitations, the trial incorporates several strengths. First, the participants were engaged during the scale development phase, to refine both the outcome measure and intervention content, enhancing comprehensibility. One way of bolstering intervention’s fidelity was by closely aligning its content development and delivery: the same multidisciplinary experts who designed the curriculum will guide the live sessions. Third, to our knowledge no prior study has evaluated a structured multicomponent intervention targeting caregiver self-efficacy in SMA and DMD, so this trial would fill a novel gap. Fourth, we will use a content-adapted scale before starting the trial’s recruitment. Establishing the scale’s relevance in Urdu ensures that the primary outcome truly reflects local caregiving contexts. Fifth, a fully digital delivery will be a major advantage in Pakistan’s context, as it increases the interventions reach nationwide, at low cost. Even the World Health Organization encourages leveraging mobile and internet platforms to extend health education in resource-limited settings [31]. Finally, embedding recruitment in the national NMD registry would allow easier and systematic identification of eligible caregivers (an efficient approach for rare disorders).

The intervention is explicitly based on social cognitive theory, which posits that self-efficacy can be enhanced through mastery experiences, vicarious learning, verbal persuasion and emotional support [32,33]. The trial’s intervention will provide these elements: educational videos and expert sessions to build knowledge (mastery), caregiver peer interactions to provide modelling (vicarious learning), and positive reinforcement cues (audio reminders) to serve as persuasive encouragement. By targeting the three domains of self-efficacy (Information, Skills, Sourcing) assessed by the adapted scale, we hypothesize incremental gains in confidence as caregivers successfully apply new practices. This supports the theoretical plausibility of intervention to be tested in a controlled design.

Findings from this trial will be disseminated through publication in peer-reviewed scientific journals and will also be presented at relevant conferences and professional meetings focusing on neuromuscular disorders. In addition, key results may be shared with relevant stakeholder communities, including patient support organisations and the Treat-NMD registry network, to inform future caregiver support initiatives and service planning.

Any protocol amendments and their respective reasons will be transparently reported in the final manuscript and submitted to the Aga Khan University Ethics Review Committee for approval prior to implementation. This is intended to allow readers to clearly distinguish pre-specified decisions from those made post hoc. In the unlikely event that the study might be terminated early, the principal investigator will inform all participants and the ethics committee immediately. Data collected up to termination will be analyzed as feasible, with a clear statement of the decision and its impact on results. No interim changes will be made without prior ethical clearance.

This protocol outlines the methods for an exploratory trial. The trial is planned using theory-informed, digitally delivered intervention to address an important gap in caregiver support for neuromuscular disorders. It is expected to provide valuable insights for an affordable solution to improve the overall quality of care for people with rare neuromuscular disorders in resource-constrained settings.

## Supporting information

S1 Checklist SPIRIT. (DOCX)

S2 Checklist TIDieR. (DOCX)

S1 File ERC Approved protocol. (DOCX)
