# Supplementary material for: A pre-post single-arm pilot trial of a digital multicomponent intervention for self-efficacy enhancement among online support groups of primary caregivers of children with neuro-muscular disorders: SEE-NMD protocol
Source: PLoS One. 2026 Nov 6;21(11):e0349543. doi: 10.1371/journal.pone.0349543 (PMC13742967; doi:10.1371/journal.pone.0349543)
Supplement: Supplementary file 3 [file pone.0349543.s003.docx]

**Effect of a Multicomponent Support Group on Self-Efficacy of Primary Caregivers of Children with Spinal Muscular Atrophy or Duchenne Muscular Dystrophy Enrolled in Treat-NMD Registry of Pakistan**

**Principal Investigator:**

Sara Khan, Associate Professor, Department of Medicine, AKU (Neuromuscular Specialist)

Email: [sara.khan@aku.edu](mailto:sara.khan@aku.edu)

**Coinvestigators:**

Nashit Irfan Aziz, Research Associate, Department of Medicine, AKU

Email: [nashit.irfan@aku.edu](mailto:nashit.irfan@aku.edu)

Wardah Khalid, Assistant Professor, Department of Community Health Sciences, AKU

Email: [wardah.khalid@aku.edu](mailto:wardah.khalid@aku.edu)

**Protocol Version:** 4.0

**Date:** 29th September 2025

## **INTRODUCTION**

Spinal Muscular Atrophy (SMA) and Duchenne Muscular Dystrophy (DMD) are severe neuromuscular disorders that significantly impact the lives of affected children and their families, placing immense pressure on primary caregivers who often experience diminished self-efficacy due to the complexities of caregiving (1). Spinal Muscular Atrophy is a genetic disorder characterized by the degeneration of motor neurons in the spinal cord, leading to muscle weakness and atrophy (2). Duchenne Muscular Dystrophy, on the other hand, is an X-linked recessive genetic disorder characterized by progressive muscle degeneration and weakness (3). Both conditions necessitate specialized and time-consuming care, creating substantial challenges for caregivers (4). These challenges are compounded by the progressive nature of these diseases, which often leads to increased functional decline, discomfort, and respiratory dysfunction in affected children (5). The constant need for monitoring, administering medication, providing physical support, and managing medical equipment can be overwhelming and exhausting. The complex care requirements associated with SMA and DMD can also lead to social isolation, financial strain, and emotional distress for caregivers, effecting their overall quality of life and potentially reducing their confidence in their ability to manage their caregiving responsibilities (6). Therefore, understanding interventions that can bolster self-efficacy among primary caregivers of children with SMA or DMD is of paramount importance.

Self-efficacy, defined as an individual's belief in their capacity to execute behaviours necessary to produce specific performance attainments, plays a pivotal role in coping with chronic illnesses and their demanding care routines (7). When caregivers possess high self-efficacy, they are more likely to approach challenges with confidence, persevere through difficulties, and experience lower levels of stress and burnout (8). Conversely, low self-efficacy can result in feelings of helplessness, anxiety, and depression, which can ultimately compromise the quality of care provided to the child and their own well-being. Given the substantial impact of SMA and DMD on both the affected children and their caregivers, interventions aimed at enhancing self-efficacy are critical. One such intervention is participation in support groups, which provide caregivers with opportunities to share experiences, gain knowledge, and receive emotional support from others facing similar challenges (9). While traditional in-person support groups have been shown to be beneficial, they may not always be accessible to caregivers due to geographical limitations, time constraints, or transportation difficulties.

Online support groups have emerged as a promising alternative, offering convenience, accessibility, and anonymity, which may be particularly appealing to caregivers who are already burdened with numerous responsibilities and constraints (10). These online platforms can facilitate peer-to-peer support, professional guidance, and access to valuable resources, all from the comfort of one's own home. Furthermore, multicomponent support groups using online modalities can integrate various elements, such as educational videos, interactive discussions, and lectures by trained professionals like Neurologists, Nutritionists, Physiotherapists. These may be particularly effective in enhancing self-efficacy by addressing the multifaceted needs of caregivers. Considering the potential benefits of multicomponent support group for primary caregivers of children with SMA or DMD, and the increasing prevalence and duration of chronic illness, there is a need for array of supports for them (11).

Currently, there are no locally validated self-efficacy tools available for primary caregivers of children with SMA or DMD in Pakistan. This necessitates the adaptation and validation of an existing tool to accurately measure self-efficacy within this specific cultural context.

This study is designed to evaluate the effect of a multicomponent support group on the self-efficacy of primary caregivers of children with SMA or DMD enrolled in the Treat-NMD registry of Pakistan. By examining the effect of this intervention, we hope to provide evidence-based recommendations for improving the well-being of caregivers and, ultimately, the quality of care for children with SMA and DMD. The findings from this study will have significant implications for healthcare providers, support organizations, and policymakers involved in the care of individuals with neuromuscular disorders and their families.

**Research Question:**

The central research question guiding this study is: Among primary caregivers of children with SMA or DMD enrolled in the Treat-NMD registry, Pakistan (P), does a multicomponent support group (I), compared with their own baseline status (C), increase their self-efficacy score (O)?

**Objectives:**

1. To adapt the contents of a multicomponent support package for self-efficacy of primary caregivers of children with SMA or DMD enrolled in the Treat-NMD registry, Pakistan.

2. To determine the effect of a multicomponent support group on the self-efficacy scores of primary caregivers of children with SMA or DMD enrolled in the Treat-NMD registry, Pakistan.

## **METHODOLOGY**

**Study Design:**

This study will be a two-phase quantitative study design. First part of the study is to adapt an existing tool that measures self efficacy of primary caregivers. The second part of the study will involve utilising this tool to measure self-efficacy scores at baseline, where participants do not receive the intervention, and then at eight weeks after they would have received the intervention.

**Study Site/Setting:**

Participants will be recruited from the Pakistan’s Neuromuscular Dystrophy Registry, a subdivision of the Treat-NMD registry, a global network of registries for neuromuscular disorders. This provides a unique opportunity to investigate the impact of interventions on caregiver outcomes. By leveraging the data and infrastructure of the Treat-NMD registry, researchers can recruit a large and diverse sample of caregivers, track their progress over time, and evaluate the effectiveness of various interventions in a standardized and rigorous manner. The study will be conducted virtually, utilizing online platforms and communication tools to facilitate the multicomponent support group and data collection.

**Study Population:**

The study population will consist of primary caregivers of children with SMA or DMD enrolled in the Treat-NMD registry, Pakistan

**Eligibility Criteria:**

Inclusion Criteria:

1. Primary caregiver of a child diagnosed with SMA or DMD.

2. Enrolled in the Treat-NMD registry- Pakistan.

3. Access to a smartphone with internet connectivity.

Exclusion Criteria:

1. Caregivers who are currently participating in another support group intervention.

**Sample Size:**

A sample size calculation was initially performed using NCSS software, based on a one-tailed t-test with a significance level of 0.05 and desired power of 80%. Because there are no prior studies of this intervention in Pakistani SMA/DMD caregivers, we conservatively assumed a medium effect size (Cohen’s d = 0.5). This corresponds to an expected mean change of 0.5 points on a 5‑point item scale (with an assumed SD of 1.0), which represents a moderate and clinically meaningful improvement (12). Based on these parameters, a sample size of 27 was obtained. After adjusting for 10% anticipated dropout rate, the sample size was 30. However, we acknowledge that this sample size estimate is preliminary and based on limited data. As such, a key objective of this study will be to obtain a more precise estimate of the expected mean difference in self-efficacy scores following the intervention. The data collected during this study will be used to calculate a more accurate effect size, which will inform future sample size calculations for larger-scale trials (13). This approach aligns with recommendations for iterative sample size refinement in the absence of strong prior evidence (14).

**Sampling technique:**

Purposive sampling will be employed to recruit participants who meet the inclusion criteria

**Operational definitions:**

Primary caregiver: The individual (from family) who provides the majority of care and support for the child with SMA or DMD (15).

Self-efficacy: An individual's belief in their ability to succeed in specific situations or accomplish a task (18).

Pre intervention: Baseline assessment of self efficacy scoring will be taken from each participant, where they will rate each item on a 5-point Likert scale, on Zoom call.

Intervention: A multifaceted strategy will be employed. Multiple groups will be created ( 3 or 4) separate for SMA and DMD patients. Each group will include up to 10 patients caregivers, 1 neurologist, 1 physiotherapist, 1 nutritionist. Sessions with each group will integrate peer support, coaching to address the multifaceted needs of patients, and time for open questions and answer sessions with the group. The session with each group will be 1 hours duration. It will start with 5 minutes talk from each expert about the basic needs for SMA and DMD patients. Following the first session, after a few weeks gap, another similar 1 hour session will be conducted which will be open to care givers to ask questions off the specialists or other care givers. In between these 2 sessions, short videos or pictorial clips for PT, OT and nutrition care will be sent to the participants to re-enforce what is discussed in the sessions. These interventions will be delivered over a period of eight weeks.

Post intervention: After 2 months, the participants of each group will be contacted for post intervention scoring via Zoom call.

**Study Variables:**

Independent variable: Multicomponent support group intervention

Dependent variable: Caregiver self-efficacy score

Covariates: Demographic variables (caregiver age, gender, education, relationship to child, child’s diagnosis, child’s age, disease duration) and baseline self-efficacy scores.

**Recruitment Strategy:**

Recruitment will primarily rely on the Treat-NMD registry from Pakistan. During data maintenance of participants on the registry, they will be given the option to participate in the study, and those who express interest will be marked out. They will eventually be contacted by the research team, informed about the study's purpose and procedures, and screened for eligibility, once the study is approved by Institute Review Board. Any phone calls made after IRB approval, for recruitment into Treat-NMD Registry, will be invited simultaneously during the same call for screening and participation.

**Data Collection (tools and process):**

Data will be collected in two sequential phases:

1. Tool adaptation/content validation of a caregiver self-efficacy instrument adapted for the Pakistani context.
2. Evaluation of the pre–post effect of a multicomponent support group intervention using the finalized tool.

Phase 1: Adaptation and Content Validation of the DMD-CSES Tool for Use in Pakistan

Step 1: Targeted Expert Input for Finalising Intervention and Tool

We will consult external experts such as a physiotherapist, occupational therapist, nutritionist, speech therapist, 1 Neurologist, 1 Paediatric Neurologist, for targeted input on finalising the multicomponent intervention and adapting DMD-CSES tool. DMD-CSES (Caregiver Self-Efficacy Scale for Duchenne Muscular Dystrophy) is a psychometrically validated tool that measures caregiver confidence across three core domains, Information, Skills, and Sourcing (15). These experts will be asked to review the tool items and corresponding parts of our intervention/study tool, ensuring language modification is also catered.

Step 2: Caregivers feedback on Intervention and Tool

We will call a group of caregivers, obtain their Consent and they will provide feedback on the clarity, relevance, and appropriateness of the intervention content.

Step 3: Content Validity Assessment (CVI)

Following feedback from Step 1 and 2, we will assess the content validity of the adapted DMD-CSES using the Content Validity Index (CVI) method, i.e. get expert panelists to rate each item on a 4-point Likert scale.

Step 4: Translation and Back-Translation

The finalized version of the tool will undergo cross-cultural adaptation into Urdu. Bilingual individuals will independently translate the English tool into Urdu. This Urdu version will then be back-translated into English by two new bilingual translators who are blinded to the original tool.

Phase 2: Pre post intervention and data collection

Caregivers participating in the multicomponent support group will be assessed at two time points: T0 (Baseline): Prior to the intervention and T1 (Follow-up): Eight weeks after the final session of the intervention. At both time points, participants will complete the final version of the self-efficacy tool.

In addition to self-efficacy scores, baseline data will also include demographic details like caregivers age, gender, education, location, relationship to child, child’s diagnosis (DMD or SMA), child’s age, and disease duration.

All data will be collected electronically. Given possible literacy and accessibility barriers, most data will be collected via structured cell phone (video) interviews conducted by trained study staff. If needed, responses will be entered into secure online forms by the staff on behalf of participants. Verbal informed consent will be obtained and documented at the start of each call. Researchers will read aloud the consent form, and the participant’s agreement will be taken.

**Quality Assurance:**

The DMD-CSES is an already psychometrically validated tool in its original setting. In this study, we will focus on cross-cultural adaptation and content validation to ensure contextual fit for Pakistan. This includes expert review, caregiver feedback, CVI scoring, and standardized translation/back-translation. Full psychometric testing (e.g., factor analysis, test–retest reliability) is beyond the scope of this project due to sample size constraints. However, the adapted tool will undergo a structured adaptation process and will then be applied in a pre–post design to assess intervention impact.

To ensure the integrity and reliability of the collected data, rigorous quality assurance measures will be implemented throughout the study. To maintain the accuracy and consistency of data collection, a comprehensive training program will be provided to all data collectors. The training will cover the study protocol, ethical considerations, data collection procedures, and the use of data collection instruments.

To minimize errors and ensure data quality, data collectors will receive ongoing supervision and support. Regular meetings will be held to address any questions or concerns, review data collection progress, and provide feedback on data quality.

Periodic audits of data collection procedures will be conducted to identify and address any deviations from the study protocol. All completed questionnaires will be reviewed weekly for completeness and consistency (17). Any missing or inconsistent data will be followed up with the participant to obtain clarification or correction.

To safeguard the confidentiality and security of participant data, a comprehensive data management plan will be developed and implemented. All electronic data will be stored on secure servers with restricted access. Data will be anonymized to protect participant privacy.

**Plan of Analysis:**

Data will first be reviewed for completeness and consistency. Cleaning procedures will include range checks, detection of outliers, and validation of logical consistency across related variables. Where appropriate, missing data patterns will be explored, and decisions regarding imputation or case-wise exclusion will be guided by the extent and mechanism of missingness.

Descriptive statistics will be used to summarize the sociodemographic characteristics of participants, including caregiver age, gender, education level, relationship to the child, and child’s age, diagnosis, and disease duration. Continuous variables (e.g., caregiver age) will be presented using mean and standard deviation (SD) if normally distributed, or median and interquartile range (IQR) if not. Categorical variables (e.g., gender, diagnosis type) will be summarized using frequencies and percentages.

The primary outcome variable is self-efficacy score, measured pre- and post-intervention using the adapted tool. Before inferential analysis, the distribution of total self-efficacy scores and subscale scores will be assessed for normality using histograms, Q-Q plots, and the Shapiro-Wilk test.

To evaluate the effect of the intervention, pre- and post-intervention self-efficacy scores will be performed. If the difference scores (post – pre) are normally distributed, a paired t-test will compare pre- and post-intervention self-efficacy scores (18). If normality is not met, the Wilcoxon signed-rank test will be applied.

All statistical tests will be two-sided, with significance defined as p < 0.05. Analyses will be conducted using STATA Version 15.

**Ethical Considerations:**

Ethical approval will be sought from the relevant Institutional Review Board prior to the commencement of the study. Informed consent will be obtained from all participants before they are enrolled in the study. The informed consent process will involve providing participants with detailed information about the study's purpose, procedures, potential risks and benefits, and their right to withdraw from the study at any time without penalty. (19).

Confidentiality of participants' data will be maintained throughout the study. All data will be stored securely and accessed only by authorized personnel. Participants' identities will be protected through the use of pseudonyms or code numbers.

Risks to participants will be minimized. The support group may bring up intense emotion. Participants will be provided with resources to seek help, including the contact information for a crisis hotline.

Before sessions start, participants will be given a set of rules to follow, as a quick reminder on do's and dont's. Zoom host controls will remain with the host (primary team members), who will only allow one speaker at a time. In case there is a heated discussion amongst participants, de escalation strategies will come into place- one of these strategies is muting particpants and preventing them from sending messages to other participants. Furthermore, the chances of such discussion are low, as the expert panel, comprising of 6 people, will also be present on zoom call. These experts have vast counselling experience due to their clinical background.

## **References**

1. Ying K, Rostenberghe HV, Kuan G, Yusoff MHAM, Ali SH, Yaacob NS. Health-Related Quality of Life and Family Functioning of Primary Caregivers of Children with Cerebral Palsy in Malaysia. International Journal of Environmental Research and Public Health [Internet]. 2021 Feb 28 [cited 2025 Feb];18(5):2351. Available from: https://doi.org/10.3390/ijerph18052351

2. Fujak A, Haaker G. Proximal spinal muscular atrophy: current orthopedic perspective. The Application of Clinical Genetics [Internet]. Dove Medical Press; 2013 Nov 1 [cited 2025 Jun];113. Available from: https://doi.org/10.2147/tacg.s53615

3. Rad N, Cai H, Weiss MD. Management of Spinal Muscular Atrophy in the Adult Population. Muscle & Nerve [Internet]. Wiley; 2022 Feb 26 [cited 2025 Jun];65(5):498. Available from: https://doi.org/10.1002/mus.27519

4. Kauffmann EF. Parents experienced cycles of defining and managing adversity in caring for a child with a chronic progressive illness. Evidence-Based Nursing [Internet]. 1998 Jan 1 [cited 2025 Feb];1(1):28. Available from: https://doi.org/10.1136/ebn.1.1.28

5. Ruythooren F, Moens P. Spinal Muscular Atrophy Scoliosis in the Era of Background Therapies—A Review of the Literature. Journal of Clinical Medicine [Internet]. 2024 Jun 14 [cited 2025 May];13(12):3467. Available from: https://doi.org/10.3390/jcm13123467

6. Esezobor CI, Solarin A, Olagunju AT. Significant Burden and Psychological Distress Among Caregivers of Children With Nephrotic Syndrome: A Cross-Sectional Study. Canadian Journal of Kidney Health and Disease [Internet]. 2020 Jan 1 [cited 2025 May];7. Available from: https://doi.org/10.1177/2054358119898016

7. Warapornmongkholkul A, Howteerakul N, Suwannapong N, Soparattanapaisarn N. Self-efficacy, social support, and quality of life among primary family-member caregivers of patients with cancer in Thailand. Journal of Health Research [Internet]. 2018 Feb 5 [cited 2025 May];32(2):111. Available from: https://doi.org/10.1108/jhr-01-2018-012

8. Noel M, Lackey E, Labi V, Bouldin ED. Efficacy of a Virtual Education Program for Family Caregivers of Persons Living with Dementia. Journal of Alzheimer s Disease [Internet]. 2022 Feb 25 [cited 2025 Feb];86(4):1667. Available from: https://doi.org/10.3233/jad-215359

9. Geere J, Moore KJ, Omondi FO, Kifalu MK, Newton CR, Hartley S. Caring for children with physical disability in Kenya: potential links between caregiving and carers’ physical health. Child Care Health and Development [Internet]. 2012 Jul 23 [cited 2025 Feb];39(3):381. Available from: https://doi.org/10.1111/j.1365-2214.2012.01398.x

10. Khan F, Saleh F, Pathan MF. Effectiveness of Diabetes Education by Health Professionals Versus Peers on Improving Diabetes Care: A Quasi-Experimental Study. Jundishapur Journal of Health Sciences [Internet]. 2018 Dec 9 [cited 2025 Jun]; Available from: https://doi.org/10.5812/jjhs.82058

11. Henry HKM, Schor EL. Supporting Self-Management of Chronic Health Problems. PEDIATRICS [Internet]. 2015 Apr 21 [cited 2025 May];135(5). Available from: https://doi.org/10.1542/peds.2014-3431

12. Skidmore ST, Thompson B. Bias and precision of some classical ANOVA effect sizes when assumptions are violated. Behavior Research Methods [Internet]. 2012 Oct 5 [cited 2025 Feb];45(2):536. Available from: https://doi.org/10.3758/s13428-012-0257-2

13. Sim J. Should treatment effects be estimated in pilot and feasibility studies? Pilot and Feasibility Studies [Internet]. 2019 Aug 28 [cited 2025 Feb];5(1). Available from: https://doi.org/10.1186/s40814-019-0493-7

14. Copsey B, Thompson J, Vadher K, Ali U, Dutton S, Fitzpatrick R, et al. Current practice in methodology and reporting of the sample size calculation in randomised trials of hip and knee osteoarthritis: a systematic review. Osteoarthritis and Cartilage [Internet]. Elsevier BV; 2018 Apr 1 [cited 2025 Feb];26. Available from: https://doi.org/10.1016/j.joca.2018.02.555

15. Türken A, Çapar H, ÇAKMAK C, KURT ME, Menteş N. Development of the self-efficacy scale in caregivers of Duchenne muscular dystrophy patients. Work [Internet]. 2023 Dec 22 [cited 2025 Jun];78(2):431. Available from: https://doi.org/10.3233/wor-230145

16. Price A, Zhu A, Nguyen HNJ, Suárez DC, Schreurs N, Burley J, et al. Study protocol for the Healthier Wealthier Families (HWF) pilot randomised controlled trial: testing the feasibility of delivering financial counselling to families with young children who are identified as experiencing financial hardship by community-based nurses. BMJ Open [Internet]. 2021 May 1 [cited 2025 May];11(5). Available from: https://doi.org/10.1136/bmjopen-2020-044488

17. Amad MA, Raja’a YA, Algendari K. Addressing dual deficiencies of SAM indicators; gaps and insights from inpatient admission to outpatient discharge, in conflict-affected Yemen; a retrospective study. Conflict and Health [Internet]. 2025 Apr 24 [cited 2025 May];19(1). Available from: https://doi.org/10.1186/s13031-025-00666-0

18. Savundranayagam MY, Brintnall-Peterson M. Testing Self-Efficacy as a Pathway That Supports Self-Care Among Family Caregivers in a Psychoeducational Intervention. Journal of Family Social Work [Internet]. 2010 Mar 19 [cited 2025 Jun];13(2):149. Available from: https://doi.org/10.1080/10522150903487107

19. Hulbert‐Williams NJ, Norwood S, Gillanders D, Finucane A, Spiller J, Strachan J, et al. Brief Engagement and Acceptance Coaching for Community and Hospice Settings (the BEACHeS Study): Protocol for the development and pilot testing of an evidence-based psychological intervention to enhance wellbeing and aid transition into palliative care. Pilot and Feasibility Studies [Internet]. 2019 Aug 20 [cited 2025 Feb];5(1). Available from: https://doi.org/10.1186/s40814-019-0488-4
