# Supplementary material for: A pre-post single-arm pilot trial of a digital multicomponent intervention for self-efficacy enhancement among online support groups of primary caregivers of children with neuro-muscular disorders: SEE-NMD protocol
Source: PLoS One. 2026 Nov 6;21(11):e0349543. doi: 10.1371/journal.pone.0349543 (PMC13742967; doi:10.1371/journal.pone.0349543)
Supplement: Supplementary file 4 [file pone.0349543.s004.docx]

Reviewer comments

**Overall Recommendation**: **Major Revision**

This is an important and timely study protocol addressing an understudied area-improving caregiver self-efficacy among caregivers of children with SMA and DMD in a low-resource setting. The protocol is generally well organized, follows SPIRIT principles, and the intervention is clearly described. However, there are several important methodological issues that should be addressed before publication.

**Major comments**

**1. Study design is weak**

The use of a single-arm pre-post design substantially limits causal inference. Improvements in self-efficacy may reflect: maturation, Hawthorne effect, regression to the mean, secular trends

The discussion acknowledges this limitation, but the protocol should better justify why a randomized controlled trial or controlled before-after design was not feasible.

**2. Sample size justification**

The sample size calculation raises concerns.

The protocol assumes: one-tailed hypothesis, Cohen's d=0.5,clinically meaningful, improvement of 0.5 points. However:

There is no empirical justification for choosing d=0.5.

One-tailed testing is rarely recommended in clinical research.

Since unexpected worsening could occur, a two-tailed test would be statistically more appropriate. The rationale should be strengthened.

**3. Outcome measurement**

The manuscript proposes adaptation of the Turkish DMD Caregiver Self-Efficacy Scale.

However: translation, cultural adaptation, caregiver interviews, content validation all appear to occur within the same protocol.

The authors should clarify:

Will validation be completed before recruitment?

Will psychometric testing include:

- internal consistency
- construct validity
- test-retest reliability
- factor analysis

Content validity alone is insufficient for adopting a new scale.

**4. Statistical analysis**

The statistical plan is incomplete.

Consider adding:

effect size (Cohen's d)

95% confidence intervals

handling of missing data

intention-to-treat versus per-protocol analysis

sensitivity analyses

Merely replacing dropouts with new participants introduces bias.

**5. Missing feasibility outcomes**

Since this is essentially a pilot intervention, feasibility outcomes should be reported, including: recruitment rate, retention, intervention adherence, attendance, participant satisfaction,acceptability

These outcomes are more informative than efficacy alone in pilot studies.

**6. Intervention fidelity**

Although standardized scripts are mentioned, additional details are needed:

Who will monitor fidelity?

Will sessions be recorded?

How will adherence to educational videos be assessed?

How will reminder exposure be confirmed?

**7. Selection bias**

Participants are recruited exclusively from a national registry.

These caregivers may be:

more engaged

more health literate

better connected

This limits generalizability.

The limitation deserves greater emphasis.

**Minor comments**

**Introduction**

The introduction is comprehensive but somewhat lengthy.

Several paragraphs discussing caregiver burden could be condensed.

**Literature**

Consider citing newer systematic reviews (2024–2026) regarding:

digital caregiver interventions

telehealth

caregiver self-efficacy

Language edits

Several grammatical issues were noted.

Examples:

"main stay of treatment"

→ "mainstay of treatment"

"comprise of"

→ "comprise"

"participants responses"

→ "participants' responses"

"study's outcome"

Use consistently:

"study outcome"

Consistency

The manuscript alternates between:

Neuro Muscular Disorders

Neuromuscular Disorders

Use one form consistently throughout.

Trial registration

Registration is appropriately reported.

However, specify whether registration occurred before enrollment

**Ethics**

Verbal consent is acceptable, but explain:

why written consent is not feasible

how documentation will occur

**Data sharing**

The manuscript states that data will not be publicly available.

PLOS ONE strongly encourages open data. The authors should explain any restrictions more clearly or indicate how anonymized data will be shared.

**Overall assessment**

This protocol addresses an important clinical and public health question and has the potential to contribute meaningfully to caregiver support research in low- and middle-income countries. However, several methodological issues—including justification of the study design, sample size assumptions, validation of the outcome measure, statistical analysis plan, and handling of missing data—require clarification before publication.

Recommendation to the Editor: **Major Revision**.
