# Supplementary material for: A pre-post single-arm pilot trial of a digital multicomponent intervention for self-efficacy enhancement among online support groups of primary caregivers of children with neuro-muscular disorders: SEE-NMD protocol
Source: PLoS One. 2026 Nov 6;21(11):e0349543. doi: 10.1371/journal.pone.0349543 (PMC13742967; doi:10.1371/journal.pone.0349543)
Supplement: Supplementary file 5 [file pone.0349543.s006.docx]

I re-reviewed the revised manuscript **PONE-D-26-21640R1**, including the revised methods and responses to the reviewers. My overall assessment is that the authors have addressed several important concerns, but **substantive methodological and editorial issues remain**. As a PLOS ONE reviewer, I would recommend **Major Revision** rather than acceptance at this stage.

**Recommendation: Major Revision**

The revised manuscript addresses several of the previous concerns, particularly clarification of the exploratory nature of the study, inclusion of effect sizes and confidence intervals, handling of withdrawals, and description of the scale-adaptation process. However, important inconsistencies and methodological issues remain.

**Major concerns**

1. **Major inconsistency in sample size**

The manuscript contains conflicting sample-size statements. The original/revised sections report **40 participants for each condition (SMA and DMD)**, whereas the revised abstract contains a visible statement of **“430 primary caregivers.”** This must be corrected throughout the manuscript. The final protocol should clearly state the total planned sample and the planned allocation between SMA and DMD.

2. **Sample-size calculation requires clarification**

The revised manuscript appears to contain remnants of two different sample-size calculations: one based on a two-tailed test with 34 participants and another based on a one-tailed calculation yielding 27 participants and 30 after dropout adjustment. The authors should retain **one internally consistent calculation**, explicitly specify the statistical test, effect size, assumed SD/correlation for the paired analysis, alpha, power, and anticipated attrition. The justification for analysing SMA and DMD separately should also be explained.

3. **The primary outcome instrument remains a significant concern**

The DMD-CSES was originally developed for DMD caregivers, while this study includes caregivers of both **DMD and SMA**. The authors have appropriately described translation, cognitive debriefing and expert content validation, but content validity alone does not establish measurement validity in SMA caregivers. The authors should explicitly state that conclusions concerning the scale's validity in SMA caregivers will remain preliminary. If feasible, they should also report reliability separately for DMD and SMA participants rather than treating the adapted instrument as fully validated.

4. **The intervention description contains internal inconsistencies**

The revised text states that participants will be delivered in groups of **10**, while other portions of the manuscript describe different batch sizes. The frequency and duration of live sessions also require reconciliation. The intervention timetable should be presented in one definitive table, including number/duration of sessions, educational materials, reminders, moderation, and expected participant exposure.

5. **Adherence/engagement should be operationally defined**

The manuscript states that intervention fidelity and delivery/read indicators will be monitored, but it does not provide sufficiently clear quantitative definitions of adherence. The authors should prespecify measures such as percentage of educational materials viewed, percentage of live sessions attended, proportion of reminders received/read, and criteria for adequate intervention exposure.

6. **Statistical analysis remains somewhat limited**

The revised plan appropriately includes effect sizes and confidence intervals. However, the authors should specify how missing baseline or post-intervention outcome data will be handled for the primary analysis. “All available data” is insufficiently precise for a prespecified protocol. They should also clarify whether the primary analysis will include all participants with paired measurements and whether analyses by diagnosis (SMA vs DMD) are exploratory.

7. **Protocol versus manuscript presentation**

The submitted PDF appears to contain substantial **track-change/revision artifacts and contradictory text**, for example phrases such as “will undertake”/“undertook,” “two-tailed”/“one-tailed,” and overlapping deleted and inserted wording. These should be completely removed from the clean manuscript before publication. The final submission must contain a coherent, final version rather than residual tracked changes.

**Minor concerns**

- The terminology should be standardized: use **“neuromuscular”** rather than alternating forms such as “neuro-muscular.”
- Several grammatical and typographical errors remain, particularly in the Methods and intervention sections.
- The statement that the adapted scale “ensures” reliability is too strong; reliability will only be **estimated** after baseline administration.
- The Discussion should avoid implying effectiveness because the single-arm design cannot distinguish intervention effects from temporal effects, regression to the mean, or Hawthorne effects.
- The protocol should clearly distinguish **feasibility outcomes** from efficacy/effectiveness outcomes.
- The data-availability statements appear inconsistent: one section states that relevant data will be available after completion, while another says the policy is not applicable and data will be available upon reasonable request. This should be harmonized.
- The ethics section should use consistent wording regarding whether approval **“was granted”** or **“was sought.”** The manuscript currently contains both formulations.

**Overall assessment**

The study addresses an **important and relatively understudied problem**, and the digital intervention is potentially relevant to caregivers of children with SMA/DMD in Pakistan. The authors have made meaningful revisions in response to previous comments. Nevertheless, the **sample-size inconsistencies, outcome-measure concerns, intervention-description inconsistencies, incomplete prespecification of missing-data analysis, and extensive residual tracked-change text** prevent me from recommending acceptance in its current form.

**Recommendation to the Editor: Major Revision.**

If the authors correct these issues carefully and provide a clean, internally consistent protocol, the manuscript could become suitable for further consideration by PLOS ONE.

Top of Form

Bottom of Form
