# Supplementary material for: A pre-post single-arm pilot trial of a digital multicomponent intervention for self-efficacy enhancement among online support groups of primary caregivers of children with neuro-muscular disorders: SEE-NMD protocol
Source: PLoS One. 2026 Nov 6;21(11):e0349543. doi: 10.1371/journal.pone.0349543 (PMC13742967; doi:10.1371/journal.pone.0349543)
Supplement: Supplementary file 6 [file pone.0349543.s007.docx]

Dear Reviewers and Editor(s)- Thank you so much for your time and timely feedback. It really helped us transform our manuscript into reflect a much better version than the initial draft.

I have divided my responses to all review comments, by labelling them with A), B), C) for Reviewer 1,2, and 3 respectively.

-------------------------------------------------------------------------------------------------------------------------------

**A) Reviewer #1-**

*Page 8 (Introduction) – Somewhere in the first paragraph, suggest that you provide some data on the costs associated with Spinal Muscular Atrophy (SMA) and Duchenne Muscular Dystrophy (DMD):

Response: Cost burden is different for each country-specific, so providing global references might not help much. We re-investigated existing Literature for cost burden of SMA/DMD, on average, in Pakistan but couldn't find a suitable reference to weave in.

*Page 13, lines 179 and 180 (Methods, Recruitment) – Suggest that you tell the reader what specific characteristics you are referring to here.

Response: This is an overlook from our end. I have deleted that line. What I meant to say by specific characteristics was the Eligibility Criteria related part.

*Page 19, lines 309 and 311 (Methods, Data Management) – Suggest that you provide a list of the do’s and don’t that you mention here as well as describing the proposed methods for de-escalating problems.

Response: Thank you for this helpful suggestion. We have expanded the manuscript to provide examples of the participant "do's and don'ts," including respectful communication, confidentiality, avoidance of abusive language and misinformation, and protection of personal information. We have also described the proposed de-escalation procedures, including private communication with participants, reminders of group rules, temporary moderation of participation, and removal from the group in the event of repeated inappropriate behaviour.

*Page 19, lines 315-334 (Methods, Statistical Analysis) – First, rather than your proposed approach to account for dropouts and nonresponse, why not conduct both per-protocol analysis, i.e., efficacy analysis, to see if the intervention worked in those who completed the entire study, as well as some type of intention-to-treat analysis, i.e., effectiveness analysis, to see if the intervention worked, regardless of whether the participants completed the study? Second, and in addition to statistical tests of significance, suggest that you establish a minimal clinically important difference (MCID) for overall scale-score changes in self-efficacy. Third, in addition to p-values, suggest that you also report 95% confidence intervals for changes in overall self-efficacy as well as the three subdomain scores for self-efficacy. Fourth, what about examining the relationship between variables such as age, sex, diagnosis type, etc., on changes in self-efficacy? Finally, suggest that you tell the reader that you will report both absolute and relative, i.e., percent changes for self-efficacy scores.

Response: The Statistical Analysis section has been substantially revised. We have clarified the approach to handling missing data and participant withdrawals, removed the proposal to replace participants after enrolment, and specified that analyses will be conducted using all available participant data where appropriate. We have also added reporting of effect sizes (Cohen's d), 95% confidence intervals, and clarified that baseline characteristics will be described using appropriate descriptive statistics. Given the exploratory nature of this pilot study and the absence of established clinical thresholds for the adapted caregiver self-efficacy scale, a minimal clinically important difference (MCID) has not been predefined. Furthermore, owing to the limited sample size, analyses examining associations between participant characteristics and changes in self-efficacy are considered exploratory and are beyond the primary objectives of this study. Lastly, percentage change, on a summed Likert score is not epidemiological standard for pre-post study design, and is difficult to interpret clinically as they do not represent ratio-level measurements with a true zero. For eg an increase from 20 to 30 and from 60 to 70 both represent a 10-point improvement, yet their percentage changes (50% vs. 16.7%) differ substantially despite the same absolute gain.

*Page 20, lines 336 through 349 (Methods, Ethical considerations) – In either this section or another, what about adverse events and how they will be handled?

Response: Since the intervention is for caregivers, mostly educational in nature, involving no physical procedures, medication, or invasive assessment, it carries a fundamentally different risk profile than a clinical or drug trial for which formal adverse event (AE) reporting frameworks are typically designed. All live sessions and moderated peer interactions are conducted in the presence of the same multidisciplinary team who designed the intervention, including clinically trained professionals with counselling experience, who are positioned to identify and respond to participant distress in real time rather than through retrospective AE reporting. Structured Q&A opportunities are embedded within the intervention itself (Week 4 pre-submission of questions, Week 5 live session), giving caregivers a routine, built-in channel to raise concerns as they arise, rather than relying on a separate formal reporting pathway. We have already specified concrete mitigation for the one plausible risk pathway identified for this delivery format i.e hostile or heated exchange during group interaction, including participant ground rules, host-level moderation controls, and immediate de-escalation by trained staff. Given the low-risk, non-invasive nature of the intervention throughout all synchronous contact points, we do not consider a separate, formal AE monitoring and reporting infrastructure proportionate to the study's risk profile.

*Page 20 (Methods, Ethical considerations) – After this section, please add a section called “Amendments to a priori protocol” where you say that any post hoc changes to your a priori protocol will be described along with the reasons for such in the final manuscript.

Response: Instead of creating a new section, we have merged it with our existing sentence that talked about documentation for changes to protocol in the Discussion section.

*Page 21, lines 367-369 (Discussion) – It would seem like tracking adherence would be relatively easy to do.

Response: Attendance throughout the intervention will be monitored and reported, and the revised changes reflect it consistently across the manuscript (aims, discussion etc). Added intervention fidelity in the section Intervention.

**B) Reviewer #2-**

*The limitations of the single-arm pre-post design should be acknowledged more explicitly in the Methods.

Response: We have revised the Methods sections by providing a clearer justification for the exploratory single-arm pre–post design and acknowledging its limitations with respect to causal inference, in response to this comment. We further clarify that this preliminary study is intended to generate feasibility data and inform the design of a future adequately powered controlled trial.

*The approach to handling participant dropouts and missing data requires clarification, as replacing participants after enrolment may introduce bias.

Response: The Statistical Analysis section has been revised to clarify the approach to participant withdrawals and missing data. The previous statement regarding replacement of participants after enrolment has been removed. Instead, we clarify that all available participant data will be analyzed where appropriate, while participant withdrawals and missing data will be transparently reported.

*The manuscript should provide more information on how intervention fidelity and participant adherence will be monitored throughout the study.

Response: We have expanded the Methods section to describe the procedures used to promote intervention fidelity and participant adherence.

*The data availability statement should be made consistent with the later description of data sharing.

Response: The Data Availability Statement has been revised, clarifying that being a protocol article, there’s no data to report; therefore, the data availability policy is not applicable.

**C) Reviewer #3-**

*The use of a single-arm pre-post design substantially limits causal inference. Improvements in self-efficacy may reflect: maturation, Hawthorne effect, regression to the mean, secular trends. The discussion acknowledges this limitation, but the protocol should better justify why a randomized controlled trial or controlled before-after design was not feasible.

Response: We agree that a single-arm pre-post design has inherent limitations regarding causal inference and have already acknowledged these limitations in the Discussion. To better justify our design choice, we have revised the Study Design section. Specifically, we now clarify that this study is intended as an exploratory evaluation of the first structured caregiver self-efficacy intervention for caregivers of children with SMA/DMD in Pakistan. Given the rarity of these disorders and the absence of locally derived effect size estimates, a preliminary single-arm study was considered an appropriate first step. The findings will provide empirical estimates of intervention effects and variability to inform the design of a future adequately powered randomized controlled trial.

*The sample size calculation raises concerns.

The protocol assumes: one-tailed hypothesis, Cohen's d=0.5,clinically meaningful, improvement of 0.5 points. However:

There is no empirical justification for choosing d=0.5.

One-tailed testing is rarely recommended in clinical research.

Since unexpected worsening could occur, a two-tailed test would be statistically more appropriate. The rationale should be strengthened.

Response: We have now strengthened the sample size section, by recalculating it with two-tailed t-test and by clarifying that no previous intervention studies evaluating caregiver self-efficacy among caregivers of children with SMA/DMD were identified; therefore, a moderate standardized effect size (Cohen's *d* = 0.5), consistent with Cohen's conventional benchmarks, was assumed for this exploratory study.

*The manuscript proposes adaptation of the Turkish DMD Caregiver Self-Efficacy Scale.

However: translation, cultural adaptation, caregiver interviews, content validation all appear to occur within the same protocol. The authors should clarify:

Will validation be completed before recruitment?

Will psychometric testing include: internal consistency, construct validity, test-retest reliability, factor analysis. Content validity alone is insufficient for adopting a new scale.

Response: We have clarified the scale adaptation process to indicate that translation, cross-cultural adaptation, caregiver cognitive debriefing, and expert content validation will be completed prior to participant recruitment, ensuring that all participants complete the finalized English and Urdu versions of the instrument. We have also clarified that internal consistency of the adapted scale will be assessed using Cronbach's alpha/McDonald's omega. We respectfully note that the objective of the present study is to adapt a validated caregiver self-efficacy scale for contextual use in Pakistan, rather than a full validation study. This is also a limitation of our study and has been stated in discussion now. Moreover, with the estimated sample size of this trial, advanced psychometrics analyses are not adequately powered for measurements.

*The statistical plan is incomplete. Consider adding:

effect size (Cohen's d), 95% confidence intervals, handling of missing data, intention-to-treat versus per-protocol analysis, sensitivity analyses. Merely replacing dropouts with new participants introduces bias.

Response: Statistical Analysis section has been substantially revised. Specifically, we have incorporated reporting of effect sizes (Cohen's *d*) and 95% confidence intervals and clarified the approach to handling missing data and participant withdrawals. The previous statement regarding replacement of participants after enrolment has been removed, as we agree that this could introduce bias. Instead, all available participant data will be analysed where appropriate, and participant withdrawals will be reported transparently. Given the exploratory nature, small sample size, and single-arm pre–post design of this pilot study, formal intention-to-treat, per-protocol, and sensitivity analyses were not considered appropriate and are therefore planned for future adequately powered controlled trials.

*Since this is essentially a pilot intervention, feasibility outcomes should be reported, including: recruitment rate, retention, intervention adherence, attendance, participant satisfaction, acceptability. These outcomes are more informative than efficacy alone in pilot studies.

Response: The manuscript has been revised to include key feasibility outcomes as secondary outcomes, including participant recruitment, retention, attendance, and participant satisfaction. We agree that these measures are important for evaluating the practicality of the intervention and informing future definitive trials.

*Although standardized scripts are mentioned, additional details are needed:

Who will monitor fidelity? Will sessions be recorded? How will adherence to educational videos be assessed? How will reminder exposure be confirmed?

Response: The Methods section has been expanded to clarify procedures for maintaining intervention fidelity. Intervention delivery will be monitored by the primary study team through standardized educational materials, facilitator guidance, and structured session content. Attendance records and reminder message dissemination will be documented throughout the intervention to monitor participant exposure.

*Participants are recruited exclusively from a national registry. These caregivers may be:

more engaged, more health literate, better connected. This limits generalizability.

The limitation deserves greater emphasis.

Response: We believe this concern arose from insufficient description of the Treat-NMD Pakistan Registry in the original manuscript. We have therefore clarified the registry enrollment process. The registry is populated through systematic ascertainment of genetically confirmed SMA/DMD cases following referral for diagnostic genetic testing at Aga Khan University Hospital, rather than through a voluntary initiative. Furthermore, all eligible registry participants will be approached for recruitment into the study, therefore recruitment is unlikely to preferentially select caregivers based on healthcare-seeking behaviour. Nevertheless, we acknowledge that participation remains dependent on successful contact, informed consent, and willingness to participate in online intervention, which may modestly limit the generalizability of the findings.

*The introduction is comprehensive but somewhat lengthy. Several paragraphs discussing caregiver burden could be condensed.

Response: The Introduction has been revised to improve conciseness by condensing the discussion on caregiver burden, while preserving the essential background and rationale for the study.

*Consider citing newer systematic reviews (2024–2026) regarding: digital caregiver interventions, telehealth, caregiver self-efficacy, Language edits needed. Several grammatical issues. Specify whether registration occurred before enrollment.

Response: The Introduction has been updated to incorporate recent reviews as suggested. Language edits corrected. Grammatical issues are also fixed. Registration was prospectively done, before enrollment in the trial, however we tweaked the wording to better reflect what we assumed was present previously in the manuscript: its date of registration was already in the abstract.

*Verbal consent is acceptable, but explain: why written consent is not feasible, how documentation will occur.

Response: We have revised the Recruitment and Ethical Considerations sections to clarify that written informed consent is not feasible because participants are geographically dispersed across Pakistan and recruitment is conducted remotely via telephone prior to enrolment in the online intervention. We have also clarified that verbal informed consent will be documented in accordance with the co-investigating institution's ethics-approved standard operating procedures for remote consent using a standardized consent log.

*The manuscript states that data will not be publicly available. PLOS ONE strongly encourages open data. The authors should explain any restrictions more clearly or indicate how anonymized data will be shared.

Response: As this manuscript describes a study protocol, no study data have yet been generated; therefore, a public dataset is not currently applicable. To address the reviewer's concern, we have stated that anonymized data generated during the study will be considered for sharing upon reasonable request following completion of the study, subject to ethical approval and data protection requirements.
