# Supplementary material for: A pre-post single-arm pilot trial of a digital multicomponent intervention for self-efficacy enhancement among online support groups of primary caregivers of children with neuro-muscular disorders: SEE-NMD protocol
Source: PLoS One. 2026 Nov 6;21(11):e0349543. doi: 10.1371/journal.pone.0349543 (PMC13742967; doi:10.1371/journal.pone.0349543)
Supplement: Supplementary file 7 [file pone.0349543.s008.docx]

I re-reviewed the revised manuscript, including the authors’ responses to the previous reviewers. Overall, the major methodological issues have been substantially addressed, but a few points still need attention before publication.

**Manuscript title**

**“A Pre-Post Single-Arm Pilot Trial of a Digital Multicomponent Intervention for Self-Efficacy Enhancement among Online Support Groups of Primary Caregivers of Children with Neuromuscular Disorders: SEE-NMD Protocol”**

**Reviewer Comments**

**Major comments**

1. **Sample size and design:** The manuscript now consistently describes 40 caregivers per condition (80 total), analyzed separately for SMA and DMD. However, the rationale for treating these as two independently powered cohorts should be stated more explicitly in the Methods and Sample Size sections.
2. **Outcome instrument:** The authors appropriately acknowledge that the DMD-CSES was originally developed for DMD caregivers and that its validity in SMA caregivers remains preliminary. Reporting internal consistency separately for the SMA and DMD groups, as planned, should be retained.
3. **Intervention consistency:** The intervention is now described in considerable detail, but the number and duration of live sessions should be made completely consistent throughout the manuscript and Figure 2. The Methods currently refer to three sessions for adherence, while the detailed schedule appears to describe different session arrangements.
4. **Statistical analysis:** The analysis plan is generally appropriate for an exploratory single-arm pilot. The handling of missing paired observations is now clearly specified. However, the manuscript should avoid language suggesting that statistical significance will establish **effectiveness**, because the absence of a control group limits causal interpretation.
5. **Data availability:** The statements regarding data availability should be carefully checked once more to ensure that the submission system, manuscript, and protocol all use identical wording appropriate for a protocol study.

**Minor comments**

- Correct remaining grammatical and typographical errors.
- Use **“neuromuscular”** consistently throughout.
- Ensure the clean manuscript contains no residual tracked changes or contradictory text.
- Consider replacing phrases such as **“ensures”** with more cautious wording when discussing validity or applicability of the adapted scale.
- Ensure Figure 2 exactly matches the intervention schedule described in the Methods.

**Overall recommendation**

**Minor revision.** The major concerns raised in the previous review have largely been addressed. The remaining issues are mainly **internal consistency, methodological wording, and clarification of the intervention and statistical terminology** rather than fundamental flaws in the protocol.
