# Supplementary material for: A pre-post single-arm pilot trial of a digital multicomponent intervention for self-efficacy enhancement among online support groups of primary caregivers of children with neuro-muscular disorders: SEE-NMD protocol
Source: PLoS One. 2026 Nov 6;21(11):e0349543. doi: 10.1371/journal.pone.0349543 (PMC13742967; doi:10.1371/journal.pone.0349543)
Supplement: Supplementary file 8 [file pone.0349543.s009.docx]

Thank you for reviewing our revised manuscript. We apologize for any lack of clarity in our last responses. To easily track our edits this time, we request the reviewers to match our responses in reference to the pages and/or line numbers quoted in the track-changes version of the manuscript.

REVIEWER 1 COMMENTS

*Page 3 (Introduction) –I think it’s important to take a more holistic approach and point out the global financial burden associated with SMA and DMD, all while pointing out that you’re not aware of any cost data associated with Pakistan.

Changes made in line 64-72, page 3,4 Introduction.

*Pages 17-18 (Methods, Data Analysis) – First, in response to my previous comments, you now state that this is a pilot/preliminary study, something that was not clearly pointed out in the original manuscript. Assuming this is true, please add this to the title. Second, while you are correct that percent change as calculated for continuous data is not appropriate for ordinal data, I was suggesting alternative approaches for non-parametric statistics that evaluate things such as rank shifts, relative position, or categorical transitions beyond what you already proposed.

Thank you for pointing it out, agreed, it was missed out initially but now we have incorporated it. We have added the word Pilot to the title and elsewhere now. Other complementary non-parametric approaches like categorical transition/relative position could make subgroup sample sizes smaller, and not give stable estimates, so we will not be putting them in for this pilot study. However, as specified in the Methods section, lines 358-359, under heading Data Analysis on page 16, Wilcoxon signed-rank would be used if normality is violated in the dataset.

REVIEWER 2 COMMENTS

1. Major inconsistency in sample size: The manuscript contains conflicting sample-size statements. The original/revised sections report 40 participants for each condition (SMA and DMD), whereas the revised abstract contains a visible statement of “430 primary caregivers.” This must be corrected throughout the manuscript. The final protocol should clearly state the total planned sample and the planned allocation between SMA and DMD.

Thank you for this comment. It is consistent now, and the 430 glitch might have been due to track changes option on word. We have rechecked this and made changes consistently now.

2. Sample-size calculation requires clarification: The revised manuscript appears to contain remnants of two different sample-size calculations: one based on a two-tailed test with 34 participants and another based on a one-tailed calculation yielding 27 participants and 30 after dropout adjustment. The authors should retain one internally consistent calculation, explicitly specify the statistical test, effect size, assumed SD/correlation for the paired analysis, alpha, power, and anticipated attrition. The justification for analysing SMA and DMD separately should also be explained.

We calculated the sample size using a two-tailed t-test and clarified that since no previous interventional studies evaluated caregiver self-efficacy among caregivers of children with SMA/DMD, a moderate standardized effect size (Cohen's *d* = 0.5), consistent with Cohen's conventional benchmarks, was assumed. We have rechecked to remove any discrepancy. All of this is present on page 7, line 143-155.

3. The primary outcome instrument remains a significant concern: The DMD-CSES was originally developed for DMD caregivers, while this study includes caregivers of both DMD and SMA. The authors have appropriately described translation, cognitive debriefing and expert content validation, but content validity alone does not establish measurement validity in SMA caregivers. The authors should explicitly state that conclusions concerning the scale & validity in SMA caregivers will remain preliminary. If feasible, they should also report reliability separately for DMD and SMA participants rather than treating the adapted instrument as fully validated.

Thankyou. Lines 400-403 now state that since DMD-CSES was originally developed and validated for DMD caregivers, conclusions regarding the scale's validity and performance among SMA caregivers will remain preliminary, pending dedicated psychometric evaluation

4. The intervention description contains internal inconsistencies: The revised text states that participants will be delivered in groups of 10, while other portions of the manuscript describe different batch sizes. The frequency and duration of live sessions also require reconciliation. The intervention timetable should be presented in one definitive table, including number/duration of sessions, educational materials,reminders, moderation, and expected participant exposure.

The revised text stated it will be given in batches of 13-14 instead of 10; again same track changes issue in the earlier version we believe. However, we anticipate these residual changes and markups won’t be a problem, if the reviewers look at the track changes version only. Furthermore, we have consolidated intervention in a suggested format, under Fig 2, ( a more definitive table).

5. Adherence/engagement should be operationally defined: The manuscript states that intervention fidelity and delivery/read indicators will be monitored, but it does not provide sufficiently clear quantitative definitions of adherence. The authors should prespecify measures such as percentage of educational materials viewed, percentage of live sessions attended, proportion of reminders received/read, and criteria for adequate intervention exposure.

Lines 214-215, and lines 261-263, have added that reminder prompts will be confirmed via in-group polling, in addition to the application's message delivery/read indicators where available. Given the exploratory nature of this trial, no adherence threshold will be pre-specified; these metrics will instead just be reported descriptively.

6. Statistical analysis remains somewhat limited: The revised plan appropriately includes effect sizes and confidence intervals. However, the authors should specify how missing baseline or post-intervention outcome data will be handled for the primary analysis. “All available data” is insufficiently precise for a prespecified protocol. They should also clarify whether the primary analysis will include all participants with paired measurements and whether analyses by diagnosis (SMA vs DMD) are exploratory.

This protocol describes two parallel, independently powered pre-post trials, one in caregivers of children with SMA and one in caregivers of children with DMD, each with its own primary analysis of change in self-efficacy. SMA and DMD data will not be pooled, and no formal statistical comparison between the two conditions is planned; each condition constitutes its own complete primary analysis rather than a subgroup or exploratory stratum of a single combined trial. We have revised the manuscript to state this explicitly. Separately, we have specified that each condition's primary analysis will include all participants with paired baseline and post-intervention DMD-CSES scores; participants missing either assessment will be excluded from that condition's paired comparison but retained in descriptive/feasibility reporting, with no imputation planned given the small per-condition sample size.

7. Protocol versus manuscript presentation: The submitted PDF appears to contain substantial track-change/revision artifacts and contradictory text, for example phrases such as “will undertake”/ “undertook,” “two-tailed”/“one-tailed,” and overlapping deleted and inserted wording. These should be completely removed from the clean manuscript before publication. The final submission must contain a coherent, final version rather than residual tracked changes.

Thank you for this comment. We have made the necessary changes

8. The terminology should be standardized: use “neuromuscular” rather than alternating forms such as “neuro-muscular.”

Incorporated in the manuscript

9. Several grammatical and typographical errors remain, particularly in the Methods and intervention sections.

Corrected

10. The statement that the adapted scale “ensures” reliability is too strong; reliability will only be estimated after baseline administration.

The word reliability has now been removed. So the statement now reads scales relevance in Urdu ensures..(line 412).

11. The Discussion should avoid implying effectiveness because the single-arm design cannot distinguish intervention effects from temporal effects, regression to the mean, or Hawthorne effects.

Our primary objective remains effectiveness, although preliminary (line 108). Whilst authors agree that single arm designs have these limitations, they have already been acknowledged in the manuscript on line 391.

12. The protocol should clearly distinguish feasibility outcomes from efficacy/effectiveness outcomes.

Stated in line 257-260.

13. The data-availability statements appear inconsistent: one section states that relevant data will be available after completion, while another says the policy is not applicable and data will be available upon reasonable request. This should be harmonized.

Thank you for this comment, it is now made consistent. We meant to say that this statement of availability isn’t applicable to protocol, as it doesn’t generate any data. However, post completion of the actual trial, data availability statements will be added.

14. The ethics section should use consistent wording regarding whether approval “was granted” or “was sought.” The manuscript currently contains both formulations.

Corrected in line 365 to “was taken”.
