# Supplementary material for: A pre-post single-arm pilot trial of a digital multicomponent intervention for self-efficacy enhancement among online support groups of primary caregivers of children with neuro-muscular disorders: SEE-NMD protocol
Source: PLoS One. 2026 Nov 6;21(11):e0349543. doi: 10.1371/journal.pone.0349543 (PMC13742967; doi:10.1371/journal.pone.0349543)
Supplement: Supplementary file 9 [file pone.0349543.s010.docx]

**Re-review: PONE-D-26-21640R3**

**Overall assessment**

The revised manuscript is substantially improved. The authors have addressed the principal concerns regarding the separate SMA and DMD cohorts, intervention scheduling, cautious interpretation of outcomes, and data-availability wording. The manuscript now explicitly acknowledges that the DMD Caregiver Self-Efficacy Scale (DMD-CSES) was developed for DMD caregivers and that its applicability to SMA caregivers remains preliminary.

The protocol addresses a relevant gap in caregiver support for rare neuromuscular disorders in Pakistan. The digital intervention is described in considerable detail, and the single-arm pilot design is reasonable for an exploratory feasibility study.

I would recommend acceptance.

**Assessment of the authors’ responses to the previous review**

| **Previous concern** | **Assessment** |
| --- | --- |
| Separate sample sizes and analyses for SMA and DMD | Addressed; the rationale for disease-specific cohorts is now explicit. |
| Validity of DMD-CSES in SMA caregivers | Addressed in the limitations; separate internal-consistency reporting should be retained. |
| Consistency of intervention sessions | The authors specify three sessions: two in Week 1 and one in Week 5. This appears resolved in the narrative, although final figure consistency should be verified. |
| Statistical significance versus intervention effectiveness | Addressed conceptually; the absence of a comparator is acknowledged. |
| Data-availability statement | The revised wording is appropriate for a protocol, but final submission fields should be checked for exact consistency. |
| Grammar, terminology, and residual tracked changes | The response indicates corrections, but some residual text and typographical issues remain in the supplied manuscript material. |

Recommendation

Acceptance
