# Supplementary material for: A pre-post single-arm pilot trial of a digital multicomponent intervention for self-efficacy enhancement among online support groups of primary caregivers of children with neuro-muscular disorders: SEE-NMD protocol
Source: PLoS One. 2026 Nov 6;21(11):e0349543. doi: 10.1371/journal.pone.0349543 (PMC13742967; doi:10.1371/journal.pone.0349543)
Supplement: Supplementary file 10 [file pone.0349543.s011.docx]

Thank you for reviewing our revised manuscript once again.

REVIEWER 3

1. Sample size and design: The manuscript now consistently describes 40 caregivers per condition (80 total), analyzed separately for SMA and DMD. However, the rationale for treating these as two independently powered cohorts should be stated more explicitly in the Methods and Sample Size sections.

We have now added the rationale for the two-cohort design explicit from lines 135-139 in the tracked document file.

2. Outcome instrument: The authors appropriately acknowledge that the DMD-CSES was originally developed for DMD caregivers and that its validity in SMA caregivers remains preliminary. Reporting internal consistency separately for the SMA and DMD groups, as planned, should be retained.

We have retained the plan to report internal consistency separately for the SMA and DMD groups.

3. Intervention consistency: The intervention is now described in considerable detail, but the number and duration of live sessions should be made completely consistent throughout the manuscript and Figure 2. The Methods currently refer to three sessions for adherence, while the detailed schedule appears to describe different session arrangements.

We have standardized the live-session description throughout the manuscript and Figure 2: the intervention includes three live sessions in total, two in Week 1 (30-35 minutes each) and one in Week 5 (60 minutes). This is now stated in the Intervention overview, the Week 1 and Week 5 descriptions, the Outcome section (adherence metrics) and Figure 2.

Week 1 includes five video clips: one introductory video about the research, which is not part of the interventional content, and **four** educational clips (two before each live session). We have clarified this in the Week 1 description and updated 5 to 4 clips for intervention, in Figure 2.

4. Statistical analysis: The analysis plan is generally appropriate for an exploratory single-arm pilot. The handling of missing paired observations is now clearly specified. However, the manuscript should avoid language suggesting that statistical significance will establish effectiveness, because the absence of a control group limits causal interpretation.

We agree that, without a control group, statistical significance cannot establish effectiveness. We have also revised causal language ("effect of the intervention", "effectiveness") in the Abstract, Introduction, Data Analysis and Discussion, to describe change in self-efficacy following the intervention.

5. Data availability: The statements regarding data availability should be carefully checked once more to ensure that the submission system, manuscript, and protocol all use identical wording appropriate for a protocol study.

We have revised the data availability statement so that the wording is identical everywhere indicated.

6. Correct remaining grammatical and typographical errors.

Corrected.

7. Use “neuromuscular” consistently throughout.

Corrected.

8. Ensure the clean manuscript contains no residual tracked changes or contradictory text.

Corrected.

9. Consider replacing phrases such as “ensures” with more cautious wording when discussing validity or applicability of the adapted scale.

Thankyou. Agreed and sure.

10. Ensure Figure 2 exactly matches the intervention schedule described in the Methods.

Rechecked and revised.
