# Supplementary material for: A pre-post single-arm pilot trial of a digital multicomponent intervention for self-efficacy enhancement among online support groups of primary caregivers of children with neuro-muscular disorders: SEE-NMD protocol
Source: PLoS One. 2026 Nov 6;21(11):e0349543. doi: 10.1371/journal.pone.0349543 (PMC13742967; doi:10.1371/journal.pone.0349543)
Supplement: Peer review history

# Peer review: Decision Letter 0

**DOI:** 10.1371/journal.pone.0349543.r001

**Authors:** Andrea Martinuzzi

21 Jul 2026

Dear Dr. Aziz,

Thank you for submitting your manuscript to PLOS ONE. After careful consideration, we feel that it has merit but does not fully meet PLOS ONE’s publication criteria as it currently stands. Therefore, we invite you to submit a revised version of the manuscript that addresses the points raised during the review process.

Please address carefully all the reviewers comments, both in terms of language curation od methodological clarity.

Please submit your revised manuscript by Aug 20 2026 11:59PM. If you will need more time than this to complete your revisions, please reply to this message or contact the journal office at plosone@plos.org. When you're ready to submit your revision, log on to https://www.editorialmanager.com/pone/ and select the 'Submissions Needing Revision' folder to locate your manuscript file.

- A letter that responds to each point raised by the academic editor and reviewer(s). You should upload this letter as a separate file labeled 'Response to Reviewers'.
- A marked-up copy of your manuscript that highlights changes made to the original version. You should upload this as a separate file labeled 'Revised Manuscript with Track Changes'.
- An unmarked version of your revised paper without tracked changes. You should upload this as a separate file labeled 'Manuscript'.

If you would like to make changes to your financial disclosure, please include your updated statement in your cover letter. Guidelines for resubmitting your figure files are available below the reviewer comments at the end of this letter.

If applicable, we recommend that you deposit your laboratory protocols in protocols.io to enhance the reproducibility of your results. Protocols.io assigns your protocol its own identifier (DOI) so that it can be cited independently in the future. For instructions see: https://journals.plos.org/plosone/s/submission-guidelines#loc-laboratory-protocols. Additionally, PLOS ONE offers an option for publishing peer-reviewed Lab Protocol articles, which describe protocols hosted on protocols.io. Read more information on sharing protocols at https://plos.org/protocols?utm_medium=editorial-email&utm_source=authorletters&utm_campaign=protocols.

As the corresponding author, your ORCID iD is verified in the submission system and will appear in the published article. PLOS supports the use of ORCID, and we encourage all coauthors to register for an ORCID iD and use it as well. Please encourage your coauthors to verify their ORCID iD within the submission system before final acceptance, as unverified ORCID iDs will not appear in the published article. *Only*  the individual author can complete the verification step; PLOS staff *cannot*  verify ORCID iDs on behalf of authors.

We look forward to receiving your revised manuscript.

Kind regards,

Andrea Martinuzzi

Academic Editor

PLOS One

Journal Requirements:

When submitting your revision, we need you to address these additional requirements.

1. Please ensure that your manuscript meets PLOS ONE's style requirements, including those for file naming. The PLOS ONE style templates can be found at

https://journals.plos.org/plosone/s/file?id=wjVg/PLOSOne_formatting_sample_main_body.pdf and

https://journals.plos.org/plosone/s/file?id=ba62/PLOSOne_formatting_sample_title_authors_affiliations.pdf.

2. Your ethics statement should only appear in the Methods section of your manuscript. If your ethics statement is written in any section besides the Methods, please delete it from any other section.

3. If the reviewer comments include a recommendation to cite specific previously published works, please review and evaluate these publications to determine whether they are relevant and should be cited. There is no requirement to cite these works unless the editor has indicated otherwise.

[Note: HTML markup is below. Please do not edit.]

Reviewers' comments:

Reviewer's Responses to Questions

**Comments to the Author**

1. Does the manuscript provide a valid rationale for the proposed study, with clearly identified and justified research questions?

Reviewer #1: Yes

Reviewer #2: Yes

Reviewer #3: Yes

**********

2. Is the protocol technically sound and planned in a manner that will lead to a meaningful outcome and allow testing the stated hypotheses?

Reviewer #1: No

Reviewer #2: Partly

Reviewer #3: Yes

**********

3. Is the methodology feasible and described in sufficient detail to allow the work to be replicable?

Reviewer #1: Yes

Reviewer #2: Yes

Reviewer #3: Yes

**********

4. Have the authors described where all data underlying the findings will be made available when the study is complete?

The PLOS Data policy

Reviewer #1: No

Reviewer #2: Yes

Reviewer #3: Yes

**********

5. Is the manuscript presented in an intelligible fashion and written in standard English?

Reviewer #1: No

Reviewer #2: Yes

Reviewer #3: Yes

**********

Please use the space provided to explain your answers to the questions above and, if applicable, provide comments about issues authors must address before this protocol can be accepted for publication. You may also include additional comments for the author, including concerns about research or publication ethics.

You may also provide optional suggestions and comments to authors that they might find helpful in planning their study.

Reviewer #1: GENERAL COMMENTS

Thank you for the opportunity to review manuscript #PONE-D-26-21640 titled “A Pre-Post Single-Arm Trial of a Multicomponent Intervention for Self-Efficacy

Enhancement among an Online Support Group of Primary Caregivers of Children with

Neuro Muscular Disorders: SEE-NMD Protocol.” I have several comments and suggestions detailed in my specific comments that follow, that I believe will strengthen this protocol. Also, some improvement in the scientific writing of this manuscript in the English-language is needed. Finally, a statement on how the data will be made available is needed.

SPECIFIC COMMENTS

*Page 8 (Introduction) – Somewhere in the first paragraph, suggest that you provide some data on the costs associated with Spinal Muscular Atrophy (SMA) and Duchenne Muscular Dystrophy (DMD).

*Page 13, lines 179 and 180 (Methods, Recruitment) – Suggest that you tell the reader what specific characteristics you are referring to here.

*Page 19, lines 309 and 311 (Methods, Data Management) – Suggest that you provide a list of the do’s and don’t that you mention here as well as describing the proposed methods for deescalating problems.

*Page 19, lines 315-334 (Methods, Statistical Analysis) – First, rather than your proposed approach to account for dropouts and nonresponse, why not conduct both per-protocol analysis, i.e., efficacy analysis, to see if the intervention worked in those who completed the entire study, as well as some type of intention-to-treat analysis, i.e., effectiveness analysis, to see if the intervention worked, regardless of whether the participants completed the study? Second, and in addition to statistical tests of significance, suggest that you establish a minimal clinically important difference (MCID) for overall scale-score changes in self-efficacy. Third, in addition to p-values, suggest that you also report 95% confidence intervals for changes in overall self-efficacy as well as the three subdomain scores for self-efficacy. Fourth, what about examining the relationship between variables such as age, sex, diagnosis type, etc., on changes in self-efficacy? Finally, suggest that you tell the reader that you will report both absolute and relative, i.e., percent changes for self-efficacy scores.

*Page 20, lines 336 through 349 (Methods, Ethical considerations) – In either this section or another, what about adverse events and how they will be handled?

*Page 20 (Methods, Ethical considerations) – After this section, please add a section called “Amendments to a priori protocol” where you say that any post hoc changes to your a priori protocol will be described along with the reasons for such in the final manuscript.

*Page 21, lines 367-369 (Discussion) – It would seem like tracking adherence would be relatively easy to do.

END OF REVIEW

Reviewer #2: This protocol addresses an important and underexplored area of caregiver support for children with neuromuscular disorders in a resource-limited setting. The rationale is clear, the intervention is well described, and the protocol follows relevant reporting guidelines. Overall, the study is feasible and has the potential to contribute meaningful evidence. However, a few issues should be addressed before publication. First, the limitations of the single-arm pre-post design should be acknowledged more explicitly in the Methods. Second, the approach to handling participant dropouts and missing data requires clarification, as replacing participants after enrolment may introduce bias. Third, the manuscript should provide more information on how intervention fidelity and participant adherence will be monitored throughout the study. Finally, the data availability statement should be made consistent with the later description of data sharing. Addressing these points will improve the methodological transparency and overall quality of the protocol.

Reviewer #3: It is good topic to select it but Please revise the manuscript based on the comments I have provided and

**********

what does this mean?). If published, this will include your full peer review and any attached files.

If you choose “no”, your identity will remain anonymous but your review may still be made public.

**Do you want your identity to be public for this peer review?**  For information about this choice, including consent withdrawal, please see our Privacy Policy

Reviewer #1: No

Reviewer #2: No

Reviewer #3: No

**********

[NOTE: If reviewer comments were submitted as an attachment file, they will be attached to this email and accessible via the submission site. Please log into your account, locate the manuscript record, and check for the action link "View Attachments". If this link does not appear, there are no attachment files.]

To ensure your figures meet our technical requirements, please review our figure guidelines: https://journals.plos.org/plosone/s/figures

You may also use PLOS’s free figure tool, NAAS, to help you prepare publication quality figures: https://journals.plos.org/plosone/s/figures#loc-tools-for-figure-preparation.

NAAS will assess whether your figures meet our technical requirements by comparing each figure against our figure specifications.

Attachment Submitted filename: Reviewer comments.docx

# Author comment: Author response to Decision Letter 1

**DOI:** 10.1371/journal.pone.0349543.r002

6 Aug 2026

Attached as a letter.

Attachment Submitted filename: Response to Reviewers.docx

# Peer review: Decision Letter 1

**DOI:** 10.1371/journal.pone.0349543.r003

**Authors:** Andrea Martinuzzi

3 Sep 2026

Dear Dr. Aziz,

Thank you for submitting your manuscript to PLOS One. After careful consideration, we feel that it has merit but does not fully meet PLOS One’s publication criteria as it currently stands. Therefore, we invite you to submit a revised version of the manuscript that addresses the points raised during the review process.

while appreciating the improvement of the revised paper, both reviewers 1 andd 3 find the work need further wìsubstantial revision.

Please submit your revised manuscript by Oct 18 2026 11:59PM. If you will need more time than this to complete your revisions, please reply to this message or contact the journal office at plosone@plos.org. When you're ready to submit your revision, log on to https://www.editorialmanager.com/pone/ and select the 'Submissions Needing Revision' folder to locate your manuscript file.

- A letter that responds to each point raised by the academic editor and reviewer(s). You should upload this letter as a separate file labeled 'Response to Reviewers'.
- A marked-up copy of your manuscript that highlights changes made to the original version. You should upload this as a separate file labeled 'Revised Manuscript with Track Changes'.
- An unmarked version of your revised paper without tracked changes. You should upload this as a separate file labeled 'Manuscript'.

If you would like to make changes to your financial disclosure, please include your updated statement in your cover letter. Guidelines for resubmitting your figure files are available below the reviewer comments at the end of this letter.

If applicable, we recommend that you deposit your laboratory protocols in protocols.io to enhance the reproducibility of your results. Protocols.io assigns your protocol its own identifier (DOI) so that it can be cited independently in the future. For instructions see: https://journals.plos.org/plosone/s/submission-guidelines#loc-laboratory-protocols. Additionally, PLOS One offers an option for publishing peer-reviewed Lab Protocol articles, which describe protocols hosted on protocols.io. Read more information on sharing protocols at https://plos.org/protocols?utm_medium=editorial-email&utm_source=authorletters&utm_campaign=protocols.

As the corresponding author, your ORCID iD is verified in the submission system and will appear in the published article. PLOS supports the use of ORCID, and we encourage all coauthors to register for an ORCID iD and use it as well. Please encourage your coauthors to verify their ORCID iD within the submission system before final acceptance, as unverified ORCID iDs will not appear in the published article. *Only*  the individual author can complete the verification step; PLOS staff *cannot*  verify ORCID iDs on behalf of authors.

We look forward to receiving your revised manuscript.

Kind regards,

Andrea Martinuzzi

Academic Editor

PLOS One

Journal Requirements:

If the reviewer comments include a recommendation to cite specific previously published works, please review and evaluate these publications to determine whether they are relevant and should be cited. There is no requirement to cite these works unless the editor has indicated otherwise.

[Note: HTML markup is below. Please do not edit.]

Reviewers' comments:

Reviewer's Responses to Questions

**Comments to the Author**

1. Does the manuscript provide a valid rationale for the proposed study, with clearly identified and justified research questions?

Reviewer #1: Yes

Reviewer #3: Yes

**********

2. Is the protocol technically sound and planned in a manner that will lead to a meaningful outcome and allow testing the stated hypotheses?

Reviewer #1: Yes

Reviewer #3: No

**********

3. Is the methodology feasible and described in sufficient detail to allow the work to be replicable?

Reviewer #1: Yes

Reviewer #3: No

**********

4. Have the authors described where all data underlying the findings will be made available when the study is complete?

The PLOS Data policy

Reviewer #1: Yes

Reviewer #3: Yes

**********

5. Is the manuscript presented in an intelligible fashion and written in standard English?

Reviewer #1: Yes

Reviewer #3: Yes

**********

Please use the space provided to explain your answers to the questions above and, if applicable, provide comments about issues authors must address before this protocol can be accepted for publication. You may also include additional comments for the author, including concerns about research or publication ethics.

You may also provide optional suggestions and comments to authors that they might find helpful in planning their study.

Reviewer #1: GENERAL COMMENTS

Thank you for the opportunity to review revised manuscript #PONE-D-26-21640R1 titled “A Pre-Post Single-Arm Trial of a Multicomponent Intervention for Self-Efficacy

Enhancement among an Online Support Group of Primary Caregivers of Children with

Neuro Muscular Disorders: SEE-NMD Protocol.” The authors have been fairly responsive to my previous comments. However, two remain, detailed below. References to different sections in my specific comments that follow are based on the marked versus unmarked manuscript. Finally, I would suggest that the authors be more specific about the location of their changes in their responses to reviewers document.

SPECIFIC COMMENTS

*Page 3 (Introduction) – I had previously suggested that you provide some data on the costs associated with Spinal Muscular Atrophy (SMA) and Duchenne Muscular Dystrophy (DMD). Your response was the following: “Cost burden is different for each country-specific, so providing global references might not help much. We re-investigated existing literature for cost burden of SMA/DMD, on average, in Pakistan but couldn't find a suitable reference to weave in.” I am well aware that the costs associated with SMA and DMD, like any disease, are country specific. The former notwithstanding, I think it’s important to take a more holistic approach and point out the global financial burden associated with SMA and DMD, all while pointing out that you’re not aware of any cost data associated with Pakistan. For example, see: Elshahawy R, Elezbawy B, Ashmawy R, et al. Global Economic Burden of Spinal Muscular Atrophy: A Systematic Literature Review. Cureus. 2025;17(3):e81023. https://doi.org/10.7759/cureus.81023

*Pages 17-18 (Methods, Data Analysis) – First, in response to my previous comments, you now state that this is a pilot/preliminary study, something that was not clearly pointed out in the original manuscript. Assuming this is true, please add this to the title. Second, while you are correct that percent change as calculated for continuous data is not appropriate for ordinal data, I was suggesting alternative approaches for non-parametric statistics that evaluate things such as rank shifts, relative position, or categorical transitions beyond what you already proposed.

END OF REVIEW

Reviewer #3: This article is good to bring to the society and I would say thank you for the authors.beside that it needs improvements

**********

what does this mean?). If published, this will include your full peer review and any attached files.

If you choose “no”, your identity will remain anonymous but your review may still be made public.

**Do you want your identity to be public for this peer review?**  For information about this choice, including consent withdrawal, please see our Privacy Policy

Reviewer #1: No

Reviewer #3: No

**********

[NOTE: If reviewer comments were submitted as an attachment file, they will be attached to this email and accessible via the submission site. Please log into your account, locate the manuscript record, and check for the action link "View Attachments". If this link does not appear, there are no attachment files.]

To ensure your figures meet our technical requirements, please review our figure guidelines: https://journals.plos.org/plosone/s/figures

You may also use PLOS’s free figure tool, NAAS, to help you prepare publication quality figures: https://journals.plos.org/plosone/s/figures#loc-tools-for-figure-preparation.

NAAS will assess whether your figures meet our technical requirements by comparing each figure against our figure specifications.

Attachment Submitted filename: I re-reviewpre-post single arm.docx

# Author comment: Author response to Decision Letter 2

**DOI:** 10.1371/journal.pone.0349543.r004

15 Sep 2026

Please match our responses in reference to the pages and/or line numbers quoted in the track-changes version of the manuscript.

A clean version labelled Manuscript is also appended so that you can look how it appears when redactions and red-lined track changes are gone.

Attachment Submitted filename: Responses to Reviewers.docx

# Peer review: Decision Letter 2

**DOI:** 10.1371/journal.pone.0349543.r005

**Authors:** Andrea Martinuzzi

29 Sep 2026

Dear Dr. Aziz,

Thank you for submitting your manuscript to PLOS One. After careful consideration, we feel that it has merit but does not fully meet PLOS One’s publication criteria as it currently stands. Therefore, we invite you to submit a revised version of the manuscript that addresses the points raised during the review process.

Please submit your revised manuscript by Nov 15 2026 11:59PM. If you will need more time than this to complete your revisions, please reply to this message or contact the journal office at plosone@plos.org. When you're ready to submit your revision, log on to https://www.editorialmanager.com/pone/ and select the 'Submissions Needing Revision' folder to locate your manuscript file.

- A letter that responds to each point raised by the academic editor and reviewer(s). You should upload this letter as a separate file labeled 'Response to Reviewers'.
- A marked-up copy of your manuscript that highlights changes made to the original version. You should upload this as a separate file labeled 'Revised Manuscript with Track Changes'.
- An unmarked version of your revised paper without tracked changes. You should upload this as a separate file labeled 'Manuscript'.

If you would like to make changes to your financial disclosure, please include your updated statement in your cover letter. Guidelines for resubmitting your figure files are available below the reviewer comments at the end of this letter.

If applicable, we recommend that you deposit your laboratory protocols in protocols.io to enhance the reproducibility of your results. Protocols.io assigns your protocol its own identifier (DOI) so that it can be cited independently in the future. For instructions see: https://journals.plos.org/plosone/s/submission-guidelines#loc-laboratory-protocols. Additionally, PLOS One offers an option for publishing peer-reviewed Lab Protocol articles, which describe protocols hosted on protocols.io. Read more information on sharing protocols at https://plos.org/protocols?utm_medium=editorial-email&utm_source=authorletters&utm_campaign=protocols.

As the corresponding author, your ORCID iD is verified in the submission system and will appear in the published article. PLOS supports the use of ORCID, and we encourage all coauthors to register for an ORCID iD and use it as well. Please encourage your coauthors to verify their ORCID iD within the submission system before final acceptance, as unverified ORCID iDs will not appear in the published article. *Only*  the individual author can complete the verification step; PLOS staff *cannot*  verify ORCID iDs on behalf of authors.

We look forward to receiving your revised manuscript.

Kind regards,

Andrea Martinuzzi

Academic Editor

PLOS One

Journal Requirements:

If the reviewer comments include a recommendation to cite specific previously published works, please review and evaluate these publications to determine whether they are relevant and should be cited. There is no requirement to cite these works unless the editor has indicated otherwise.

Please review your reference list to ensure that it is complete and correct. If you have cited papers that have been retracted, please include the rationale for doing so in the manuscript text, or remove these references and replace them with relevant current references. Any changes to the reference list should be mentioned in the rebuttal letter that accompanies your revised manuscript. If you need to cite a retracted article, indicate the article’s retracted status in the References list and also include a citation and full reference for the retraction notice.

[Note: HTML markup is below. Please do not edit.]

Reviewers' comments:

Reviewer's Responses to Questions

**Comments to the Author**

1. Does the manuscript provide a valid rationale for the proposed study, with clearly identified and justified research questions?

Reviewer #1: Yes

Reviewer #3: Yes

**********

2. Is the protocol technically sound and planned in a manner that will lead to a meaningful outcome and allow testing the stated hypotheses?

Reviewer #1: Yes

Reviewer #3: Yes

**********

3. Is the methodology feasible and described in sufficient detail to allow the work to be replicable?

Reviewer #1: Yes

Reviewer #3: Yes

**********

4. Have the authors described where all data underlying the findings will be made available when the study is complete?

The PLOS Data policy

Reviewer #1: Yes

Reviewer #3: Yes

**********

5. Is the manuscript presented in an intelligible fashion and written in standard English?

Reviewer #1: Yes

Reviewer #3: Yes

**********

Please use the space provided to explain your answers to the questions above and, if applicable, provide comments about issues authors must address before this protocol can be accepted for publication. You may also include additional comments for the author, including concerns about research or publication ethics.

You may also provide optional suggestions and comments to authors that they might find helpful in planning their study.

Reviewer #1: GENERAL COMMENTS

Thank you for the opportunity to review revised manuscript #PONE-D-26-21640R2 titled “A Pre-Post Single-Arm Trial of a Multicomponent Intervention for Self-Efficacy

Enhancement among an Online Support Group of Primary Caregivers of Children with

Neuro Muscular Disorders: SEE-NMD Protocol.” The authors have adequately responded responsive to my previous comments and suggestions. I have no other comments or suggestions.

END OF REVIEW

Reviewer #3: I want to say thank you to the authors of this manuscript for their dedicated work and to bring such interesting title. Please modify for some of our comments.

**********

what does this mean?). If published, this will include your full peer review and any attached files.

If you choose “no”, your identity will remain anonymous but your review may still be made public.

**Do you want your identity to be public for this peer review?**  For information about this choice, including consent withdrawal, please see our Privacy Policy

Reviewer #1: No

Reviewer #3: No

**********

[NOTE: If reviewer comments were submitted as an attachment file, they will be attached to this email and accessible via the submission site. Please log into your account, locate the manuscript record, and check for the action link "View Attachments". If this link does not appear, there are no attachment files.]

To ensure your figures meet our technical requirements, please review our figure guidelines: https://journals.plos.org/plosone/s/figures

You may also use PLOS’s free figure tool, NAAS, to help you prepare publication quality figures: https://journals.plos.org/plosone/s/figures#loc-tools-for-figure-preparation.

NAAS will assess whether your figures meet our technical requirements by comparing each figure against our figure specifications.

Attachment Submitted filename: I re review for single arm.docx

# Author comment: Author response to Decision Letter 3

**DOI:** 10.1371/journal.pone.0349543.r006

6 Oct 2026

document for response to reviewers updated. Please read in conjunction with track changes file and look at clean copy of the Manuscript as well. We have tried our best to incorporate the suggested changes.

many thanks for taking the time for such detailed feedbacks.

Attachment Submitted filename: Responses_to_Reviewers_auresp_3.docx

# Peer review: Decision Letter 3

**DOI:** 10.1371/journal.pone.0349543.r007

**Authors:** Andrea Martinuzzi

13 Oct 2026

A Pre-Post Single-Arm Pilot Trial of a Digital Multicomponent Intervention for Self-Efficacy Enhancement among Online Support Groups of Primary Caregivers of Children with Neuro-Muscular Disorders: SEE-NMD Protocol

PONE-D-26-21640R3

Dear Dr. Aziz,

We’re pleased to inform you that your manuscript has been judged scientifically suitable for publication and will be formally accepted for publication once it meets all outstanding technical requirements.

Within one week, you’ll receive an e-mail detailing the required amendments. When these have been addressed, you’ll receive a formal acceptance letter and your manuscript will be scheduled for publication.

An invoice will be generated when your article is formally accepted. Please note, if your institution has a publishing partnership with PLOS and your article meets the relevant criteria, all or part of your publication costs will be covered. Please make sure your user information is up-to-date by logging into Editorial Manager at Editorial Manager® and clicking the ‘Update My Information' link at the top of the page. For questions related to billing, please contact billing support.

If your institution or institutions have a press office, please notify them about your upcoming paper to help maximize its impact. If they’ll be preparing press materials, please inform our press team as soon as possible -- no later than 48 hours after receiving the formal acceptance. Your manuscript will remain under strict press embargo until 2 pm Eastern Time on the date of publication. For more information, please contact onepress@plos.org.

Kind regards,

Andrea Martinuzzi

Academic Editor

PLOS One

Additional Editor Comments (optional):

Reviewers' comments:

Reviewer's Responses to Questions

**Comments to the Author**

1. Does the manuscript provide a valid rationale for the proposed study, with clearly identified and justified research questions?

Reviewer #3: Yes

**********

2. Is the protocol technically sound and planned in a manner that will lead to a meaningful outcome and allow testing the stated hypotheses?

Reviewer #3: Yes

**********

3. Is the methodology feasible and described in sufficient detail to allow the work to be replicable?

Reviewer #3: Yes

**********

4. Have the authors described where all data underlying the findings will be made available when the study is complete?

The PLOS Data policy

Reviewer #3: Yes

**********

5. Is the manuscript presented in an intelligible fashion and written in standard English?

Reviewer #3: Yes

**********

Please use the space provided to explain your answers to the questions above and, if applicable, provide comments about issues authors must address before this protocol can be accepted for publication. You may also include additional comments for the author, including concerns about research or publication ethics.

You may also provide optional suggestions and comments to authors that they might find helpful in planning their study.

Reviewer #3: Thank you for revising the manuscript and addressing the previous reviewer comments. The revised protocol is clearer, particularly regarding the separate SMA and DMD cohorts, the intervention schedule, and the preliminary nature of applying the DMD-CSES instrument in the SMA population. The study addresses an important clinical and psychosocial need among caregivers of children with neuromuscular disorders.

**********

what does this mean?). If published, this will include your full peer review and any attached files.

If you choose “no”, your identity will remain anonymous but your review may still be made public.

**Do you want your identity to be public for this peer review?**  For information about this choice, including consent withdrawal, please see our Privacy Policy

Reviewer #3: No

**********

Attachment Submitted filename: Re-review.docx

# Editor report: Acceptance letter

**DOI:** 10.1371/journal.pone.0349543.r008

**Authors:** Andrea Martinuzzi

PONE-D-26-21640R3

PLOS One

Dear Dr. Aziz,

I'm pleased to inform you that your manuscript has been deemed suitable for publication in PLOS One. Congratulations! Your manuscript is now being handed over to our production team.

At this stage, our production department will prepare your paper for publication. This includes ensuring the following:

* All references, tables, and figures are properly cited

* All relevant supporting information is included in the manuscript submission,

* There are no issues that prevent the paper from being properly typeset

You will receive further instructions from the production team, including instructions on how to review your proof when it is ready. Please keep in mind that we are working through a large volume of accepted articles, so please give us a few days to review your paper and let you know the next and final steps.

Lastly, if your institution or institutions have a press office, please let them know about your upcoming paper now to help maximize its impact. If they'll be preparing press materials, please inform our press team within the next 48 hours. Your manuscript will remain under strict press embargo until 2 pm Eastern Time on the date of publication. For more information, please contact onepress@plos.org.

You will receive an invoice from PLOS for your publication fee after your manuscript has reached the completed accept phase. If you receive an email requesting payment before acceptance or for any other service, this may be a phishing scheme. Learn how to identify phishing emails and protect your accounts at https://explore.plos.org/phishing.

If we can help with anything else, please email us at customercare@plos.org.

Thank you for submitting your work to PLOS One and supporting open access.

Kind regards,

PLOS One Editorial Office Staff

on behalf of

Dr. Andrea Martinuzzi

Academic Editor

PLOS One
